# Identification of a putative novel genotype 3/rabbit hepatitis E virus (HEV) recombinant

**DOI:** 10.1371/journal.pone.0203618

**Published:** 2018-09-11

**Authors:** Ka-Cheung Luk, Kelly E. Coller, George J. Dawson, Gavin A. Cloherty

**Affiliations:** Infectious Disease Research, Abbott Diagnostics, Abbott Park, Illinois, United States of America; Centers for Disease Control and Prevention, UNITED STATES

## Abstract

Hepatitis E virus (HEV) is a viral pathogen transmitted by the fecal-oral route and is a major cause of waterborne acute hepatitis in many developing countries. In addition to infecting humans, HEV has been identified in swine, wild boars, rabbits and other mammals; with swine and wild boars being main reservoirs for zoonotic transmission of HEV. There are four major HEV genotypes known to infect humans; genotypes 1 (HEV-1) and 2 (HEV-2) are restricted to humans, and genotypes 3 (HEV-3) and 4 (HEV-4) are zoonotic. Herein, three human HEV strains originating in France were sequenced and near full-length genomes were characterized. Phylogenetic analysis showed that two strains were genotype 3 and closely grouped (a 100% bootstrap value) with subtype 3i reference strains. In percent nucleotide identities, these two strains were 94% identical to each other, 90–93% identical to subtype 3i strains, 82–86% identical to other HEV-3, and 77–79% identical to rabbit HEV strains excluding the two divergent strains KJ013414 and KJ013415 (74%); these two strains were less than 77% identical to strains of HEV genotypes 1, 2 and 4. The third strain was found distinct from any known HEV strains in the database, and located between the clusters of HEV-3 and rabbit HEV strains. This unique strain was 74–75% identical to HEV-1, 73% to HEV-2, 81–82% to HEV-3, 77–79% to rabbit HEV again excluding the two divergent strains KJ013414 and KJ013415 (74%), and 74–75% to HEV-4, suggesting a novel unclassified strain associated with HEV-3 and rabbit HEV. SimPlot and BootScan analyses revealed a putative recombination of HEV-3 and rabbit HEV sequences at four breakpoints. Phylogenetic trees of the five fragments of the genome confirmed the presence of two HEV-3 derived and three unclassified sequences. Analyses of the amino acid sequences of the three open reading frames (ORF1-3) encoded proteins of these three novel strains showed that some amino acid residues specific to rabbit HEV strains were found solely in this unclassified strain but not in the two newly identified genotype 3i strains. The results obtained by SimPlots, BootScans, phylogenetic analyses, and amino acid sequence comparisons in this study all together appear to suggest that this novel unclassified strain is likely carrying a mosaic genome derived from HEV-3 and rabbit HEV sequences, and is thus designated as a putative genotype 3/rabbit HEV recombinant.

## Introduction

The global burden of hepatitis E virus (HEV) is estimated to be 20 million infections yearly with 3 million people showing signs of symptomatic hepatitis [1]. HEV is a non-enveloped virus with a single-stranded, positive-sense RNA genome of approximately 7.2 kb in length [2, 3]. The HEV genome contains a 5’ untranslated region (UTR), three open reading frames (ORFs), and a 3’ UTR [4, 5]. ORF1 encodes a non-structural polyprotein containing several functional domains, including methyltransferase (MT), a Y domain, papain-like cysteine protease (PCP), a hypervariable region (HVR), a proline-rich region (Pro), an X domain, helicase (Hel), and RNA-dependent RNA polymerase (RdRp). ORF2 encodes the capsid protein, while ORF3 encodes a phosphoprotein.

Although only a single serotype has been determined [6], HEV strains belonging to the *Hepeviridae* family display extensive genetic diversity [7, 8]. A taxonomic scheme was recently proposed [9] to classify this family into two genera: *Orthohepevirus* and *Piscihepevirus*. *Orthohepevirus* contains all mammalian and avian HEV strains, and is divided into four species: *Orthohepevirus A-D*. *Orthohepevirus A* includes four HEV major genotypes (1–4, or HEV-1 to HEV-4). HEV-1 and HEV-2 are restricted to humans, and transmitted through the consumption of contaminated water. HEV-3 and HEV-4 have a wide host range including humans, swine, wild boars and other mammals, and are responsible for zoonotic transmission from animals to humans through the consumption of raw or undercooked meats in both developing and industrialized countries [10–14]. Additional *Orthohepevirus A* genotypes have been found in rabbits (HEV-3ra), wild boars in Japan (HEV-5 and HEV-6), and camels in the Middle East (HEV-7) and China (HEV-8) [15–17]. Other HEV species in the *Orthohepevirus* genus infect birds (*Orthohepevirus B*), rats, ferrets and minks (*Orthohepevirus C*), and bats (*Orthohepevirus D*) [9, 18].

Rabbit HEV strains have been found in farmed, wild, pet and laboratory rabbits in China [19, 20], the United States [21, 22], France [23, 24], Italy [25, 26], Germany [27, 28, 29], the Netherlands [30], Korea [31], and Canada [32]. Phylogenetic analysis of full-length genomic sequences indicates that all rabbit HEV strains together form a separated clade that is closely related to HEV-3 (76–79% nucleotide identities, excluding the two highly divergent rabbit-derived sequences KJ013414 and KJ013415 with only 72–73% identities), but distant from other genotypes of HEV [33]. The genomes of all rabbit HEV strains harbor a signature insertion of 93-nucleotides (nt) (31 amino acids) in the X domain of ORF1 that is absent in any other known HEV strains [34]. The presence of this insertion in rabbit HEV strains may indicate a significant difference between rabbit HEV and HEV-3. Recently, rabbit HEV infections in humans have been confirmed in France, thus providing evidence of zoonotic transmission of rabbit HEV to humans [35].

RNA-RNA recombination appears to be a common phenomenon in positive-sense RNA viruses [36–38]. Although the exchange of genetic material frequently occurs within a viral population, it can take place between two different viral strains or between two different viruses [39, 40]. RNA recombination is one of the major factors responsible for the emergence of new, often dangerous viral strains or species [41]. RNA recombination in RNA viruses is mediated by a viral replicase, RdRp, via a template-switch [39, 42]. In the proposed life cycle of HEV [43, 44], the viral RdRp generates an intermediate, replicative negative-sense RNA from the positive-sense genomic RNA that in turn serves as the template for the synthesis of positive-sense, progeny viral genomes. It is assumed that when a single cell is co-infected with two different strains of HEV, RdRp initiates nascent strand synthesis at the 3’ end of genomic RNA of one strain, pauses, dissociates, and reassociates with RNA template of another strain to resume strand synthesis to generate an HEV RNA recombinant [39, 42]. In fact, recombination in HEV has been documented [45, 46] between two different human strains, or between human and swine strains.

The emergence of new HEV recombinant forms may have implications from the perspective of screening, diagnostic testing, therapeutics and patient monitoring. To better understand the impact of genetic diversity on assay performance and to support the development of assays capable of reliably detecting all HEV strains, we are engaged in monitoring diversification of HEV and searching for newly emerging variants. HEV RNA positive samples were sourced from France (n = 9) [Discovery Life Sciences (DLS), Los Osos, California, USA], but 3 samples were not able to be genotyped by the vendor’s routine laboratory methods. In the present study, we report the sequencing and characterization of the near full-length genome sequences of these three HEV samples. Phylogenetic analysis showed that one strain was found distinct from any known HEV strains in the GenBank database and standing alone in between the clusters of HEV-3 and rabbit HEV strains. SimPlot and BootScan analyses revealed a pattern of putative recombination events between HEV-3 and rabbit HEV strains. Another two strains belonged to genotype 3 and were closely related to a wild boar HEV subtype 3i strain identified in Germany, further supporting zoonotic transmission of HEV from wild boars to humans.

## Materials and methods

### Samples and RNA extraction

HEV RNA positive human plasma samples (DLS13-11677, DLS13-11681 and DLS13-11685) were sourced from France [Discovery Life Sciences (DLS), Los Osos, California, USA]. Using the Altona RealStar HEV RT-PCR Kit, the HEV viral loads (copies/ml) determined by the vendor were 230,000 for DLS13-11677, 132,000 for DLS13-11681, and 10,900 for DLS13-11685, but their genotypes were not known. A volume of 0.6 ml plasma for each of the three samples was extracted for viral RNA on an automated *m*2000*sp* instrument using the *m*2000*sp* 0.6 ml HIV-1 RNA protocol (Abbott Molecular, Des Plaines, IL).

The epidemiological data of these three patients was largely unavailable. As based on the information provided by the vendor, all three plasma samples were collected on January 11, 2013 in France. No information on the gender, age, travel history, occupation, hobbies, eating habits, living place, or any contacts with animals was available. As a result, we have not been able to identify the routes by which these three patients became infected with HEV.

### Preparation of cDNA libraries

First-strand cDNA synthesis was performed using a SuperScript III First-Strand Synthesis System (Invitrogen, Carlsbad, CA) according to the manufacturer’s instructions. In brief, 8 μl extracted viral RNA of each sample was mixed with 1 μl of 50 ng/μl of random hexamers and 1 μl of 10 mM dNTP, and preheated at 65°C for 5 min. 10 μl of cDNA synthesis master mix containing reaction buffer and 200 units of SuperScript III polymerase was added to each 10 μl of RNA/primer mixture. Each 20 μl reaction mixture was then incubated at 25°C for 10 min which was followed by 50°C for 50 min. Second-strand cDNA synthesis was performed using Sequenase (USB Corporation, Cleveland, OH) as previously described [47]. Each cDNA library was purified, concentrated and eluted in 6 μl of DNA elution buffer using a DNA Clean and Concentrator-5 Kit (Zymo Research, Irvine, CA) per the manufacturer’s instructions.

### Next-generation sequencing (NGS) library preparation

5 μl of each purified cDNA library was added to Nextera XT reaction mixtures (Illumina, San Diego, CA). Compatible barcodes were selected, and the manufacturer’s protocol was followed, except that 16 cycles of PCR were performed instead of 12. Libraries were purified once more with AMP-Pure XP beads (Beckman Coulter, Beverly, MA) according to the manufacturer’s protocol, and eluted in 30 μl of Illumina resuspension buffer (RSB). Library concentrations were measured on a BioAnalyzer 2200 TapeStation using a D1K screen tape (Agilent Technologies, Santa Clara, CA) based on integration of peaks from 150 to 700 nucleotides (nt) and then adjusted to a 1 nM final concentration before multiplexing. Libraries were combined in equal volumes, denatured with 0.1 N (final concentration) NaOH for 5 min, and diluted to 20 pM with HT1 buffer. The multiplex library was diluted once more with HT1 to 12 pM. The multiplex library was denatured at 96°C for 2 min, chilled on ice, and then run on a MiSeq instrument using a 300-cycle MiSeq reagent kit v2 (Illumina).

### NGS analysis

Barcodes were parsed on the MiSeq instrument, and reads were filtered for Q-scores above 30. Fastq files were imported into CLC Genomics Workbench 9.0 software (CLC bio/Qiagen, Aarhus, Denmark), and Illumina paired-end reads 1 and 2 were merged. Paired-end reads were aligned repeatedly to one or more HEV complete genome sequences representing genotypes 1–4 from the GenBank database to obtain a final consensus sequence. Gaps in coverage were observed for all three HEV consensus sequences in this study. Thus, PCR primers were designed based on the known sequences obtained from the NGS to amplify and determine the missing sequences.

### PCR and dideoxy chain termination (Sanger) sequencing

PCR primers were listed in S1 Table. Synthesis of cDNA and one-round PCR amplification were performed using a QIAGEN OneStep RT-PCR Kit (Qiagen Inc., Valencia, CA). Briefly, reactions were carried out in a total volume of 50 μl, containing 0.4 μM each of forward and reverse primers, 1 x reaction buffer, 0.4 mM of each dNTP, 1x Q-Solution, 2 μl of enzyme mix, and 10 μl of extracted viral RNA. Incubations were performed in a GeneAmp 9700 thermocycler (Applied Biosystems, Foster City, CA) at 50°C for 30 min, 95°C for 15 min, followed by 50 cycles of 94°C for 30 sec, 50°C for 30 sec, 72°C for 1 min 30 sec, and a final extension of 72°C for 10 min. PCR amplified products were purified using a QIAquick Purification Kit (Qiagen Inc.) according to the manufacturer’s instructions. Dideoxy chain termination (Sanger) sequencing reactions were prepared using the Big Dye Terminator Cycle Sequencing Ready Reaction Kit v3.1 and electrophoresed on an ABI 3130xl Genetic Analyzer (Applied Biosystems). Sequencing data was analyzed using Sequencher v5.4.6 (Gene Codes Corp., Ann Arbor, MI). These Sanger sequences were then merged with the NGS data in Sequencher software to generate final near full-length genomic sequences for the three HEV strains.

### Phylogenetic analysis

To determine the genotypes, the three newly generated HEV sequences were aligned with a panel of HEV full genome sequences representing different genotypes obtained from the GenBank database using the CLUSTAL W method in MegAlign (Lasergene version 14, DNASTAR Inc., Madison, WI). Alignments were then converted into phylogenetic trees using the methods as described previously [48]. Viral sequences were individually analyzed for evidence of recombination using SimPlot (version 3.5.1; S. Ray, Johns Hopkins University, Baltimore, MD). The percent identity was calculated between the query sequence and a panel of representative sequences in a sliding window, which is moved across the alignment in steps, to identify intergenotype mosaicism. If recombination was indicated, BOOTSCAN and FINDSITE were performed, and breakpoints were confirmed by constructing phylogenetic trees for each individual fragment of the genome.

### Nucleotide sequence accession numbers

The near full-length genome sequences of these three HEV strains have been deposited in the GenBank database under the following accession numbers: MG783569 (DLS13-11677), MG783570 (DLS13-11681), and MG783571 (DLS13-11685).

## Results

### Phylogenetic classifications of HEV sequences

The three HEV samples were first examined by NGS on cDNA libraries constructed with random primers. Coverage gaps were then amplified and sequenced using PCR primers and Sanger sequencing. Analyses of the overlapping NGS reads and sequences of PCR amplification products resulted in near full-length genomic sequences of 7222, 7224, and 7191 nt, excluding the 3’-end poly(A) tails, for samples DLS13-11677, DLS13-11681, and DLS13-11685, respectively. The genomic organizations of these three HEV strains were similar to those of HEVs from other *Orthohepevirus A* species, with a 5’ UTR, followed by three open reading frames (ORF1, ORF2, and ORF3), and a 3’ UTR (Table 1).

**Table 1:** Genomic organizations of three novel HEV strains in this study.

Strain	5’ UTR (nt)*	ORF1 (nt)*	ORF2 (nt)*	ORF3 (nt)*	3’ UTR (nt)*	PolyA Site (nt)*
DLS13-11677	1–25	26–5137	5172–7154	5134–5502	7155–7222	7223
DLS13-11681	1–27	28–5139	5174–7156	5136–5504	7157–7224	7225
DLS13-11685	1–3	4–5103	5138–7123	5100–5471	7124–7191	7192

*Nucleotide (nt) position refers to the sequence of DLS13-11677 (MG783569), DLS13-11681 (MG783570), or DLS13-11685 (MG783571).

Classifications of these three new HEV sequences were first carried out by sequence comparisons with known HEV complete genome sequences in GenBank using the BLAST search tool. The top three sequences in the BLAST search revealed that both DLS13-11677 and DLS13-11681 shared a 93% nucleotide identity with a French human HEV strain KJ701409 (genotype 3i), and a 90% nucleotide identity with two other subtype 3i strains, a wild boar HEV strain identified in Germany (FJ705359) and another French human HEV strain (KU176129). In contrast, the top three HEV strains closest to DLS13-11685 were human HEV genotype 3 strains found in Japan (3e-AB248520, 3b-AB291962 and 3a-AB369689), with an 82% nucleotide sequence identity.

**Fig 1:**
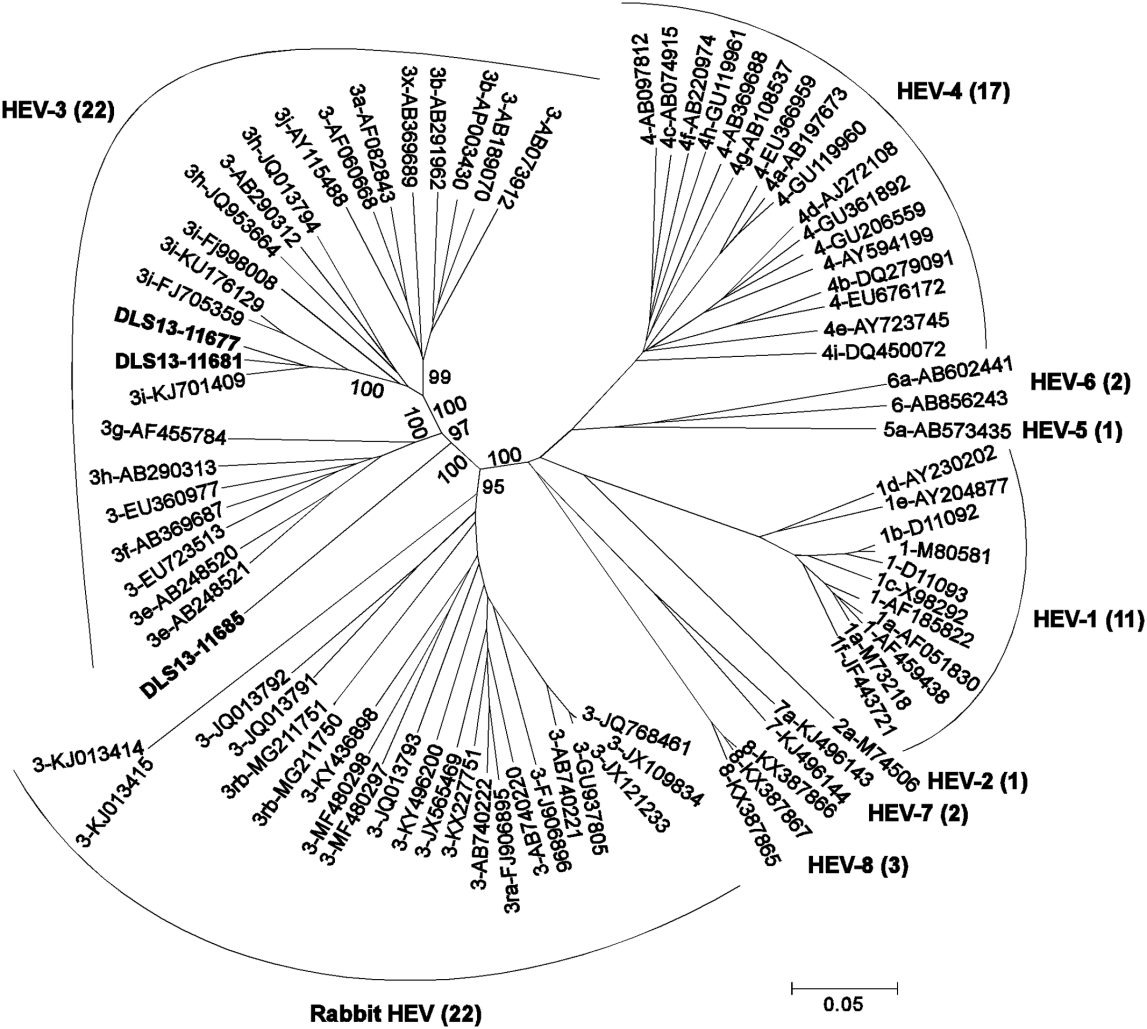
Phylogenetic tree of strains DLS13-11677, DLS13-11681 and DLS13-11685. Phylogenetic tree was constructed by aligning complete genomes of 81 HEV reference sequences (see S2 Table for subtype, GenBank accession number, and strain for each of the 81 sequences) with the genomes of DLS13-11677, DLS13-11681 and DLS13-11685. The alignment was then gap-stripped to become 6,900 nucleotides long and converted to PHYLIP format using BioEdit Sequence Alignment Editor (version 5.0.9). Phylogenetic analysis was performed with the PHYLIP software package (version 3.5c). A phylogenetic tree was constructed using TreeExplorer software (version 2.12). Genotype designations, subtypes, and GenBank accession numbers are shown above the appropriate branches. Only the relevant bootstrap values are indicated in the tree.

To better classify these three new strains, a phylogenetic analysis was performed. As based on the HEV reference sequences proposed by Smith et al. [15] and used by others [28, 29, 33, 35], 81 complete genomes of *Orthohepevirus A* species were retrieved from the GenBank database (S2 Table), including 11 HEV-1, 1 HEV-2, 22 HEV-3, 22 rabbit HEV, 17 HEV-4, 1 HEV-5, 2 HEV-6, 2 HEV-7, and 3 HEV-8 strains. A phylogenetic tree was constructed using the 81 reference sequences and the genomes of DLS13-11677, DLS13-11681, and DLS13-11685 (Fig 1). Strains DLS13-11677 and DLS13-11681 paired closely from the same branch along with the French human HEV-3i strain (KJ701409) in agreement with the BLAST search result. These three strains together with the other two genotype 3i strains (the German wild boar strain 3i-FJ705359 and another French human strain 3i-KU176129) formed a group with a high degree of confidence supported by a bootstrap value of 100% inside the cluster of genotype HEV-3. In percent nucleotide identities from the alignment (Fig 1), DLS13-11677 and DLS13-11681 were 94% identical to each other, 93% to 3i-KJ701409, 90% to 3i-FJ705359 and 3i-KU176129, 82–86% to other HEV-3, 77–79% to rabbit HEV excluding the two highly divergent rabbit-derived sequences KJ013414 and KJ013415 (a 74% identity), and 74–76% to other genotypes (HEV-1, 2, 4, 5, 6, 7 and 8).

On the contrary, the branch of DLS13-11685 in the phylogenetic tree of Fig 1 was found standing alone in between the clusters of HEV-3 (a bootstrap value of 97%) and rabbit HEV (a bootstrap value of 100%) strains, and appeared to be distinct from all known HEV strains. In percent nucleotide identities from the alignment, DLS13-11685 was 81–82% identical to HEV-3, 77–79% to rabbit HEV excluding the two highly divergent sequences KJ013414 and KJ013415 (74%), and 73–75% to other genotypes (HEV-1, 2, 4, 5, 6, 7 and 8), thus suggesting that this newly obtained third sequence was novel and unclassified, but was likely associated with both HEV-3 and rabbit HEV sequences. In contrast to the two newly identified subtype 3i strains (DLS13-11677 and DLS13-11681) which were at least 90% identical to the three reference 3i strains but only 82–86% identical to other HEV-3, this DLS13-11685 unclassified strain only had a narrow range of 81–82% identities to all HEV-3 strains tested, including the top three closest Japanese human strains with an 82% identity.

To further confirm the genotype classifications of these three strains, another phylogenetic tree was constructed by using a total of 154 HEV complete genomes (16 HEV-1, 1 HEV-2, 80 HEV-3, 22 rabbit HEV, 22 HEV-4, 1 HEV-5, 2 HEV-6, 2 HEV-7, 3 HEV-8, 2 rat HEV, DLS13-11677, DLS13-11681, and DLS13-11685), including the 81 HEV reference sequences described in Fig 1 and S2 Table, and 70 additional HEV sequences listed in S3 Table. As displayed in S1 Fig, DLS13-11677 and DLS13-11681 still closely grouped together with the two genotype 3i strains KJ701409 and FJ705359 (a bootstrap value of 100%) inside the cluster of HEV-3 strains. On the other hand, DLS13-11685 was still found standing in between the clusters of rabbit HEV (a bootstrap value of 100%) and HEV-3 (a bootstrap value of 92%) strains. These results are in agreement with those in Fig 1 having only 81 HEV reference sequences, thus confirming the 3i genotyping for DLS13-11677 and DLS13-11681, and the unclassified status for DLS13-11685.

### Characterization of recombination events

The genome of this novel and unclassified strain DLS13-11685 was first analyzed for evidence of recombination using a SimPlot software. The nucleotide percent identity was calculated between the query sequence (DLS13-11685) and a panel of five HEV reference sequences (1 HEV-1, 1 HEV-2, 1 HEV-3, 1 HEV-4, and 1 rabbit HEV) in a sliding window, which was moved across the 6,900 nt alignment of Fig 1 in steps (a segment of 400 nt with an increment of 40 nt), to identify intergenotype mosaicism. After scanning a series of panels containing different combinations of five HEV reference sequences, a clear recombination pattern was obtained in the following three panels of five sequences: (1) HEV-1 (1a-AF051830), HEV-2 (2a-M74506), HEV-3 (3e-AB248520), HEV-4 (4g-AB108537), and rabbit HEV (JQ013791), (2) HEV-1 (1a-AF051830), HEV-2 (2a-M74506), HEV-3 (3e-AB248520), HEV-4 (4g-AB108537) and rabbit HEV (KY436898), and (3) HEV-1 (1a-AF051830), HEV-2 (2a-M74506), HEV-3 (3e-AB248520), HEV-4 (4g-AB108537) and rabbit HEV (MF480298).

**Fig 2:**
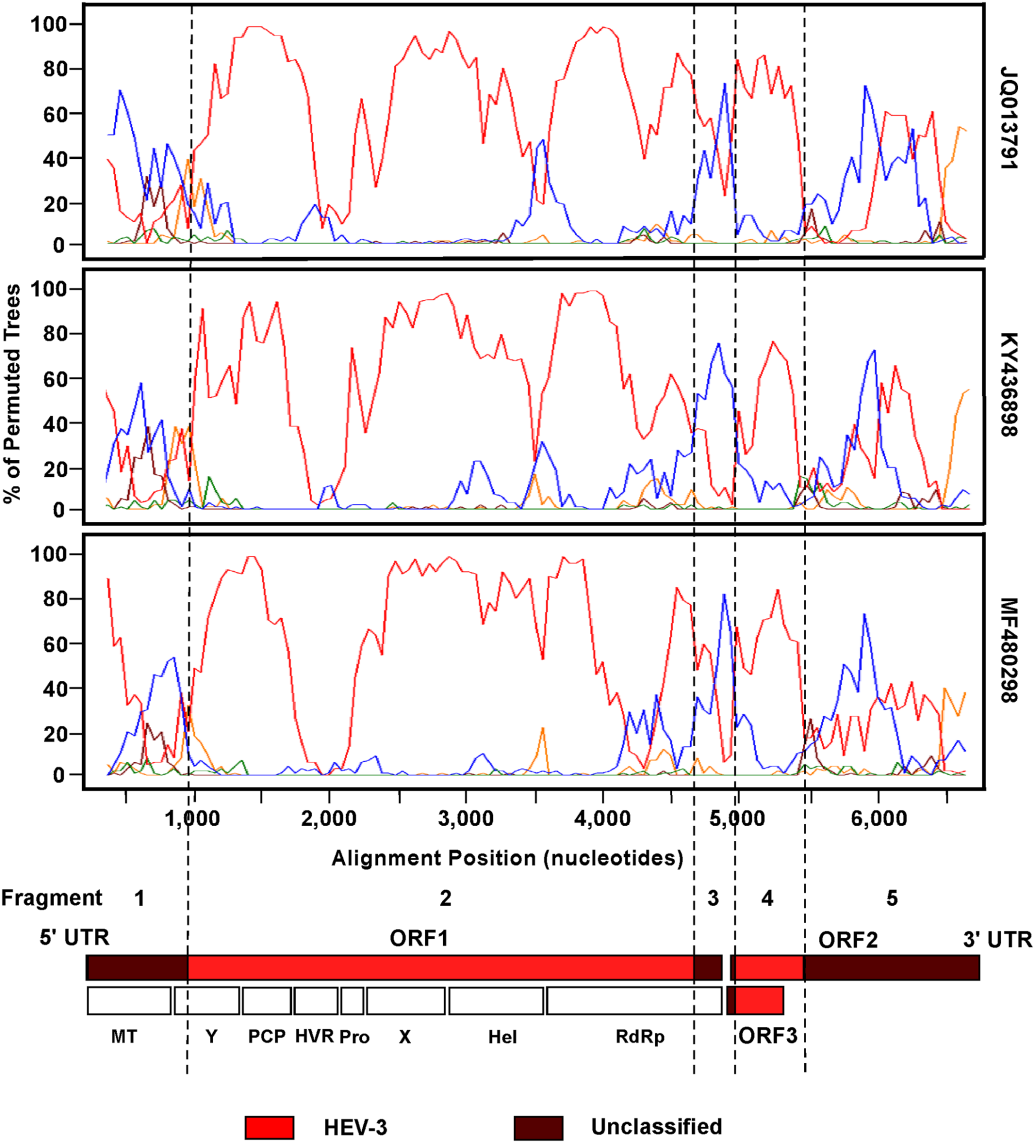
BootScans and Genetic Organization of the DLS13-11685 genome. BootScan analyses were performed on the 6,900 nt sequence alignment of Fig 1. DLS13-11685 genome was compared to five HEV reference sequences, including HEV-1 (1a-AF051830, green), HEV-2 (2a-M74506, brown), HEV-3 (3e-AB248520, red), HEV-4 (4g-AB108537, orange), and rabbit HEV (JQ013791, KY436898, or MF480298, blue). The three open reading frames (ORF1, ORF2 and ORF3) of the DLS13-11685 genome are shown below the three BootScan plots along with the approximate locations within ORF1 coding for methyltransferase domain (MT), Y domain (Y), papain-like cysteine protease (PCP), hypervariable region (HVR), proline-rich domain (Pro), X domain (X), helicase (Hel), and RNA-dependent RNA polymerase (RdRp). The vertical dashed lines denote the putative recombination breakpoints that partition the viral genome of DLS13-11685 into five fragments (1–5). Fragments 1, 3 and 5 (in brown) are designated as unclassified (containing a mixture of rabbit HEV and HEV-3 sequences), whereas fragments 2 and 4 (in red) have HEV-3 sequences.

BootScan was then performed using the same sliding window parameters as in SimPlot on the query sequence of DLS13-11685 and the three panels of the five reference sequences mentioned above. As illustrated in Fig 2, BootScan plots of DLS13-11685 generated by the three panels revealed a similar recombination pattern and mosaic genetic composition, consisting of HEV-3 (3e-AB248520) and rabbit HEV (JQ013791, KY436898, or MF480298) sequences, with four putative recombination breakpoints. Based on these four putative recombination breakpoints, the DLS13-11685 genome could be divided into five potential fragments. Fragments 1, 3 and 5 were unclassified as they seemingly contained both rabbit HEV and HEV-3 sequences. Fragments 2 and 4 were likely derived from HEV-3.

The four putative recombination breakpoints for the genome of DLS11685 were further refined using the FindSites function of SimPlot. Informative sites indicated that the four putative breakpoints of the five fragments were located at the following nt positions in the sequence of DLS13-11685 (MG783571): fragment 1 (unclassified; complete MT domain, and 5’ end of the Y domain in ORF1) extended from nt 38 to 891 (854 nt long); fragment 2 (potentially HEV-3 derived; majority of 3’ region encoding for Y, complete domains of PCP, HVR, Pro, X, and Hel, and 5’ part of RdRp encoding sequence in ORF1) from nt 892 to 4794 (3903 nt); fragment 3 (unclassified; 3’ part of RdRp encoding sequence, 5’ ends of ORF2 and ORF3) from nt 4795 to 5157 (363 nt); fragment 4 (potentially HEV-3 derived; 5’ small section of ORF2, and majority of 3’ ORF3) from nt 5158 to 5584 (427 nt); and fragment 5 (unclassified; majority of 3’ ORF2) from nt 5585 to 7093 (1509 nt).

**Fig 3:**
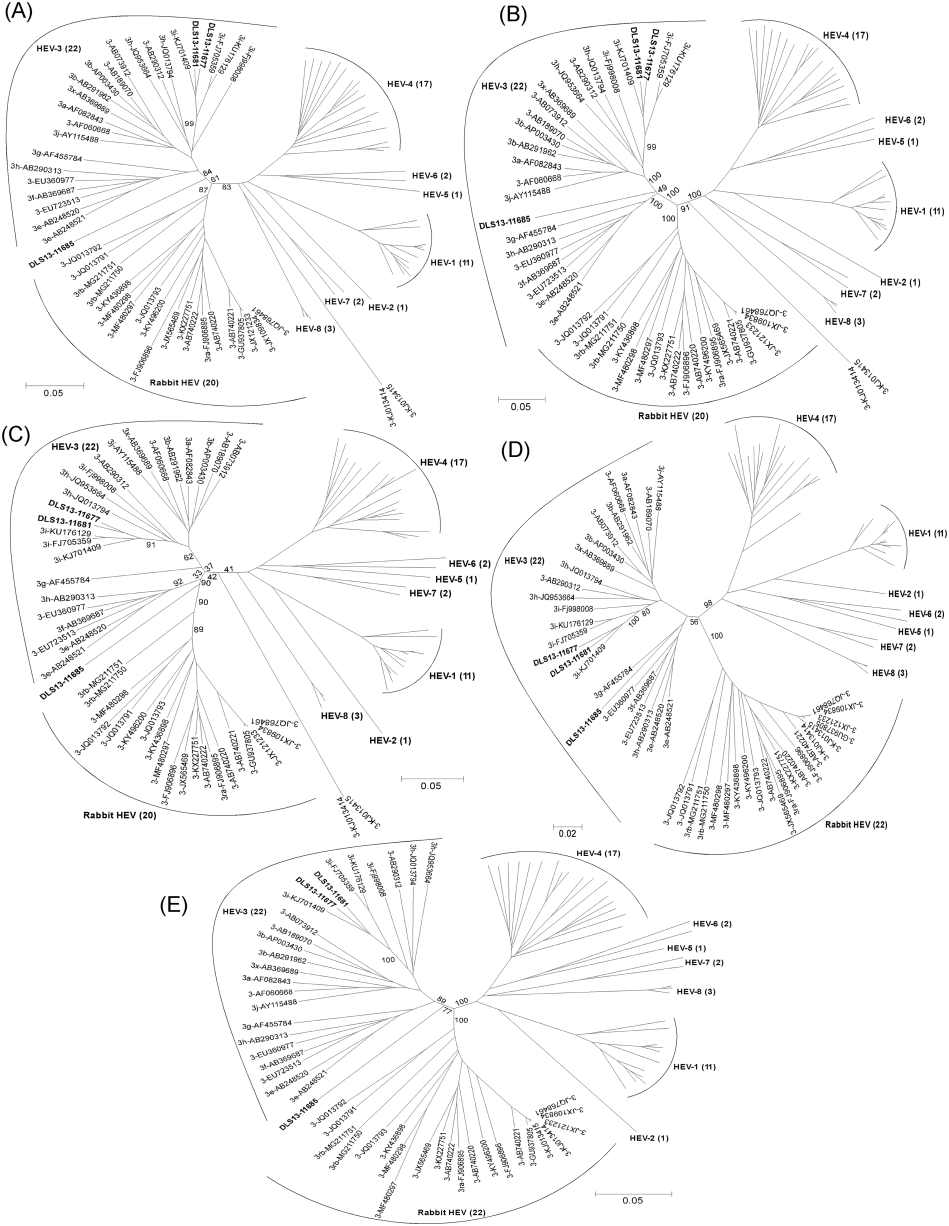
Phylogenetic trees of five individual fragments of DLS13-11685 defined by BootScan and FindSites analyses. Nucleotide sequences of (A) fragment 1 (nt 38–891; 854 nt; unclassified), (B) fragment 2 (nt 892–4794; 3903 nt; HEV-3 derived), (C) fragment 3 (nt 4795–5157; 363 nt; unclassified), (D) fragment 4 (nt 5158–5584; 427 nt; HEV-3 derived), or (E) fragment 5 (nt 5585–7093; 1509 nt; unclassified) from the alignment of Fig 1. Nucleotide positions refer to the sequence of DLS13-11685. Genotype designations for the reference strains are shown above the appropriate branches. Only the relevant GenBank accession numbers of the reference sequences and bootstrap values are shown in the trees.

To further verify the genetic composition across the genome of strain DLS13-11685 as described above, phylogenetic trees derived from the 6,900 nt sequence alignment of Fig 1 were constructed for the five individual fragments defined by BootScan and FindSites analyses (Fig 3A–3E). In fragments 1, 3 and 5 (Fig 3A, 3C and 3E), DLS13-11685 was found located in between the clusters of HEV-3 (bootstrap values of 84–90%) and rabbit HEV (bootstrap values of 61–90%) strains, suggesting that each of these three fragments might have contained both HEV-3 and rabbit HEV sequences. In nucleotide percent identities, fragment 1 was 80–81% identical to HEV-3 and 78–80% to rabbit HEV (only 73% to the two divergent strains KJ013414 and KJ013415), fragment 3 was 79–83% identical to HEV-3 and 79–81% to rabbit HEV (only 72–73% to KJ013414 and KJ013415), and fragment 5 was also 79–83% identical to HEV-3 and 78–81% to rabbit HEV (81% to KJ013414 and KJ013415). All three fragments had similar identities to HEV-3 and similar identities to rabbit HEV, and the percent identities to both HEV-3 and rabbit HEV were similar among all three fragments, thus suggesting that fragments 1, 3 and 5 were likely derived from the sequences of HEV-3 and rabbit HEV strains, and that they were unclassified. Within fragment 5 of BootScans (Fig 2), there was a potential breakpoint at nucleotide 6,000 to separate rabbit HEV sequence from HEV-3. Individual phylogenetic trees of these two sub-fragments showed that they were also unclassified similar to the unclassified status of the parental fragment 5 (data not shown). As a result, fragment 5 remained as one unclassified fragment in this study. In the phylogenetic tree of fragment 2 (Fig 3B), this fragment grouped with HEV-3 strains with a bootstrap value of 100% away from rabbit HEV and other genotype strains. In nucleotide percent identities, fragment 2 was 79–80% identical to HEV-3 and 75–77% to rabbit HEV (only 70% to KJ013414 and KJ013415). The percent identities to HEV-3 in fragment 2 were similar to those in fragments 1, 3 and 5. But the percent identities to rabbit HEV in fragment 2 were expectedly lower than those in the three fragments, thus indicating that these three fragments likely obtained some rabbit HEV sequences to raise the percent identities. Similar to fragment 2, fragment 4 (Fig 3D) also grouped with HEV-3 strains with a bootstrap value of 56% away from the rabbit HEV cluster. But the nucleotide percent identities to both HEV-3 (87–89%) and rabbit HEV (82–85%) (83% to KJ013414 and KJ013415) were higher in fragment 4 than in fragment 2. As fragment 4 (427 nt) contained most of the ORF3 sequence (315 out of 372 nt; 84.7%) (Fig 2), it is likely that the stronger selection pressure in the ORF2/ORF3 overlapping region within this fragment as compared to other genomic regions without the overlapping reading frames might have kept the sequence of the ORF2/ORF3 overlapping region largely unchanged as a native sequence of HEV-3 with high nucleotide percent identities to both HEV-3 and rabbit HEV strains.

For comparison, the five individual fragments of the genomes of the two newly identified genotype 3i strains DLS13-11677 and DLS13-11681, as in the case of the complete genomes (Fig 1), were found always closely grouping next to each other, and to the three genotype 3i reference strains (KJ701409, FJ705359, and KU176129) inside the cluster of HEV-3 strains (Fig 3A–3E), further demonstrating the genotype 3i classification for these two new genomes.

**Fig 4:**
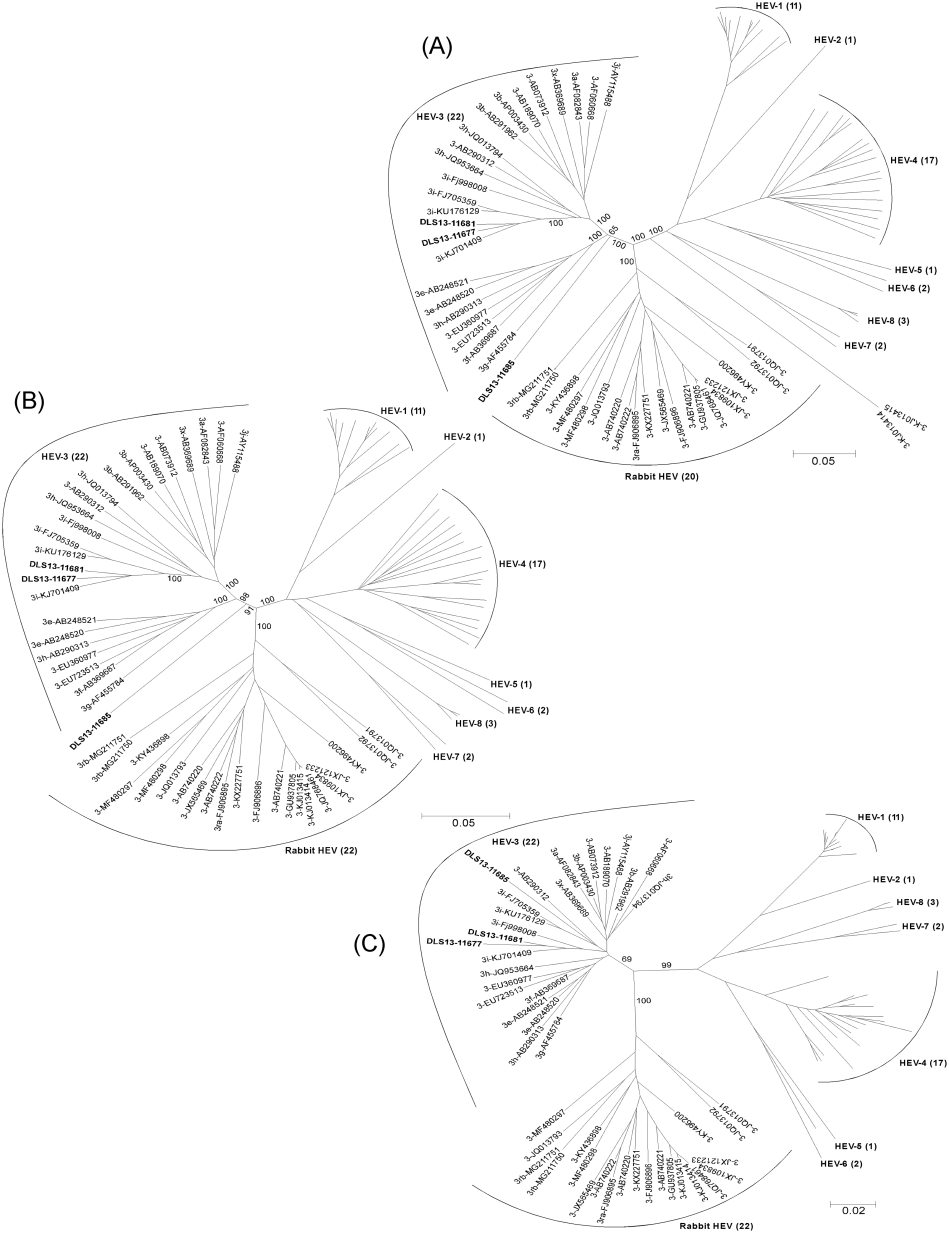
Phylogenetic trees of ORF1, ORF2 and ORF3 nucleotide sequences of DLS13-11677, DLS13-11681 and DLS13-11685. Nucleotide sequences of (A) ORF1, (B) ORF2, and (C) ORF3 of strains DLS13-11677, DLS13-11681 and DLS13-11685 were aligned with those of 81 HEV reference sequences as described in Fig 1 and S2 Table. Genotype designations and relevant GenBank accession numbers are shown above the appropriate branches. Only the relevant bootstrap values are indicated on the branches.

In addition to the complete genome nucleotide sequences, the phylogenetic trees were constructed using the nucleotide sequences of ORF1, ORF2, and ORF3 of the three newly identified strains with the same panel of 81 HEV reference strains as described in Fig 1 and S2 Table. As shown in Fig 4A–4C, DLS13-11677 and DLS13-11681, as in the case of complete genome sequences, closely grouped together with the three genotype 3i reference strains (KJ701409, FJ705359, and KU176129) inside the cluster of HEV-3 strains in all three ORF nucleotide sequences, thus keeping their genotype 3i classifications. Similar to the results of the complete genome analysis (Fig 1), DLS13-11685 remained as an unclassified strain as indicated by its position outside the HEV-3 strain cluster as well as outside the rabbit HEV strain cluster in both phylogenetic trees of ORF1 (Fig 4A) and ORF2 (Fig 4B). As illustrated in the BootScan plots (Fig 2), both ORF1 and ORF2, like the complete genome, contained HEV-3 and unclassified nucleotide sequences, which might have contributed the unclassified status for these two ORFs of DLS13-11685. Drastically different from ORF1 and ORF2, ORF3 nucleotide sequence of DLS13-11685 (Fig 4C) closely grouped with HEV-3 strains with 91–94% identities, in agreement with the HEV-3 classification of fragment 4 (Fig 3D) that contains most of the ORF3 nucleotide sequence (Fig 2).

To assess the amino acid compositions of this unclassified strain DLS13-11685, the amino acid sequences of ORF1, ORF2 and ORF3 encoded proteins from 22 HEV-3 (S2 Table), 22 rabbit HEV (S2 Table), DLS13-11677, DLS13-11681, and DLS13-11685 were aligned respectively. As shown in S4 Table and Table 2, ORF1-encoded polyprotein amino acid sequence of DLS13-11685 had 23 amino acid residues (8 in fragment 1 and 15 in fragment 2) present either exclusively or predominantly in rabbit HEV sequences but not or rarely in HEV-3. Particularly, amino acids 154, 515, 609 and 1502 (Table 2) were observed specific to rabbit HEV sequences for DLS13-11685 and to HEV-3 sequences for the two genotype 3i strains (DLS13-11677 and DLS13-11681). Similarly, ORF2-encoded capsid protein amino acid sequence of DLS13–11685 (S5 Table and Table 3) was found to have 6 amino acid residues (1 in fragment 4 and 5 in fragment 5) present either solely or mainly in rabbit HEV sequences but not or rarely in HEV-3. Amino acid 241 (Table 3), in particular, was a rabbit HEV-specific amino acid “L” for DLS13-11685, and an HEV-3 specific amino acid “I” for the two genotype 3i strains. Both fragment 3 and HEV-3 derived ORF3-encoded phosphoprotein amino acid sequence of DLS13–11685 (S6 Table) did not have any amino acids found only or predominantly in rabbit HEV sequences but not or sparsely in HEV-3. Overall, no any amino acid stretches indicating the presence of HEV-3 and rabbit HEV recombination nucleotide sequences were observed in the proteins encoded by the three ORFs of this unclassified strain.

**Table 2:** Rabbit HEV-specific amino acid residues in ORF1-encoded polyprotein of DLS13-11685.

Strain	aa 41*	aa 62	aa 152	aa 154	aa 184	aa 189
DLS13-11677	V (nonpolar/alkyl)	L (nonpolar/alkyl)	R (polar/basic)	S (polar/neutral)	T (polar/neutral)	A (nonpolar/alkyl)
DLS13-11681	V	L	R	S	T	A
DLS13-11685	T (polar/neutral)	F (nonpolar/aromatic)	G (nonpolar/alkyl)	T (polar/neutral)	A (nonpolar/alkyl)	V (nonpolar/alkyl)
HEV-3 (22)	17 V/3 T	20 L/2 F	21 R/0 G	18 A/4 S/0 T	22 T/0 A	19 A/3 V
Rabbit HEV (22)	19 T/1 V	22 F/0 L	15 G/1 R	12 T/7 A/0 S	9 A/9 T	20 V/2 A
	**aa 235**	**aa 248**	**aa 307**	**aa 515**	**aa 517**	**aa 576**
DLS13-11677	I (nonpolar/alkyl)	D (polar/acidic)	S (polar/neutral)	F (nonpolar/aromatic)	V (nonpolar/alkyl)	V (nonpolar/alkyl)
DLS13-11681	I	D	S	L (nonpolar/alkyl)	V	V
DLS13-11685	T (polar/neutral)	E (polar/ acidic)	C (polar/neutral)	S (polar/neutral)	I (nonpolar/alkyl)	F (nonpolar/aromatic)
HEV-3 (22)	22 I/0 T	22 D/0 E	22 S/0 C	18 L/0 S/2 F	21 V/1 I	16 V/0 F
Rabbit HEV (22)	6 T/15 I	5 E/17 D	4 C/18 S	13 S/0 F/0 L	8 I/14 V	4 F/5 V
	**aa 609**	**aa 622**	**aa 719**	**aa 892**	**aa 1011**	**aa 1157**
DLS13-11677	T (polar/neutral)	S (polar/neutral)	A (nonpolar/alkyl)	Q (polar/neutral)	A (nonpolar/alkyl)	I (nonpolar/alkyl)
DLS13-11681	T	S	A	Q	A	I
DLS13-11685	A (nonpolar/alkyl)	T (polar/neutral)	V (nonpolar/alkyl)	H (polar/basic)	S (polar/neutral)	V (nonpolar/alkyl)
HEV-3 (22)	19 T/0 A	20 S/1 T	22 A/0 V	20 Q/2 H	15 A/7 S	22 I/0 V
Rabbit HEV (22)	8 A/0 T	6 T/16 S	4 V/18 A	21 H/0 Q	22 S/0 A	3 V/19 I
	**aa 1502**	**aa 1506**	**aa 1540**	**aa 1594**	**aa 1645**	
DLS13-11677	E (polar/acidic)	A (nonpolar/alkyl)	V (nonpolar/alkyl)	I (nonpolar/alkyl)	I (nonpolar/alkyl)	
DLS13-11681	E	A	V	I	I	
DLS13-11685	D (polar/acidic)	S (polar/neutral)	I (nonpolar/alkyl)	V (nonpolar/alkyl)	V (nonpolar/alkyl)	
HEV-3 (22)	21 E/0 D	15 A/7 S	13 V/9 I	22 I/0 V	21 I/1 V	
Rabbit HEV (22)	22 D/0 E	22 S/0 A	21 I/0 V	19 V/3 I	15 V/4 I	

*Amino acid (aa) position refers to S4 Table.

**Table 3:** Rabbit HEV-specific amino acid residues in ORF2-encoded capsid protein of DLS13-11685.

Strain	aa 147*	aa 208	aa 241	aa 359	aa 396	aa 645
DLS13-11677	S (polar/neutral)	I (nonpolar/alkyl)	I (nonpolar/alkyl)	T (polar/neutral)	L (nonpolar/alkyl)	I (nonpolar/alkyl)
DLS13-11681	S	I	I	T	L	I
DLS13-11685	T (polar/neutral)	V (nonpolar/alkyl)	L (nonpolar/alkyl)	M (nonpolar/alkyl)	V (nonpolar/alkyl)	V (nonpolar/alkyl)
HEV-3 (22)	22 S/0 T	22 I/0 V	21 I/0 L	22 T/0 M	22 L/0 V	18 I/4 V
Rabbit HEV (22)	20 T/2 S	3 V/19 I	22 L/0 I	20 M/2 T	18 V/2 L	21 V/0 I

*Amino acid (aa) position refers to S5 Table.

The HEV viral loads (copies/ml) provided by the vendor (DLS) were 230,000 for DLS13-11677, 132,000 for DLS13-11681, and 10,900 for DLS13-11685. To investigate if the low viral load of DLS13-11685 was caused by the sequence variations between the strain and the primers/probe in the vendor’s quantitative assay, we have developed a real-time PCR HEV quantitative assay (unpublished) with a set of primers and probe in the ORF3 region which was conserved among the HEV strains including the three strains in this study. Using the WHO HEV plasma standard as a calibrator, the HEV viral loads (IU/ml) were determined to be 33,115 for DLS13-11677, 14,899 for DLS13-11681, and 2,882 for DLS13-11685, verifying the low viral titer of DLS13-11685 which was not caused by sequence variations.

## Discussion

In this study, we have sequenced and characterized near full-length genomes of three human HEV strains sourced from France. Our characterizations of the first two HEV strains DLS13-11677 and DLS13-11681 demonstrated their close relationships with two other French human HEV strains (3i-KJ701409 and 3i-KU176129), and a wild boar HEV strain wbGER27 (3i-FJ705359) identified in Germany [12]. The two new genomes obtained here shared a 93% nucleotide identity to KJ701409, and a 90% identity to either KU176129 or FJ705359, which are greater than the lower limit of identity at the level of subtype defined by Lu et al. for the HEV-3 genomes (82.0–87.9%) [49]. As a result, these two new strains are classified as genotype 3i. It has been reported [50] that consumption of some pork products, such as raw liver, was a major source of exposure for autochthonous HEV infection in France. Conceivably, it is likely that these four French genotype 3i human strains might have originated from wild boars through the route of zoonotic transmission.

In a phylogenetic tree of complete genome sequences, the third new HEV strain DLS13-11685 was found standing alone in between the clusters of HEV-3 and rabbit HEV strains, suggesting a novel unclassified strain potentially related to both HEV-3 and rabbit HEV strains. In both SimPlot and BootScan analyses, the genome of DLS13-11685 showed a similar recombination pattern at four breakpoints between a Japanese 3e strain AB248520 and one of the three rabbit HEV strains (JQ013791, KY436898, or MF480298), apparently resulting from high percent nucleotide identities between DLS13-11685 and these four strains. While 3e-AB248520 shared an 82% identity with DLS13-11685 showing up on top in the BLAST search, the three rabbit HEV strains had a range of 78–79% identities. Other genotypes (HEV-1, 2, 4, 5, 6, 7, and 8) shared a lower range of 73–75% identities. Although 3e-AB248520 was identified in Japan, rabbit HEV strain JQ013791 was from France, and the other two rabbit HEV strains KY436898 and MF480298 were found in Germany. Sample DLS13-11685 was collected in France.

Phylogenetic trees of the five fragments of the DLS13-11685 genome from the recombination patterns confirmed the presence of three unclassified (fragments 1, 3 and 5, likely containing a mixture of HEV-3 and rabbit HEV sequences) and two HEV-3 derived (fragments 2 and 4) sequences. Similarly, phylogenetic trees of the nucleotide sequences of the three ORFs of DLS13-11685 demonstrated that ORF1 and ORF2 were unclassified, while ORF3 was HEV-3 derived. Stronger selection pressure in the ORF2/ORF3 overlapping region of HEV likely might have kept the nucleotide sequences of ORF3 and fragment 4 of DLS13-11685 largely unchanged. Since both sequences have been proven belonging to HEV-3 with nucleotide identities of 87–94%, it is most likely that DLS13-11685 might have started out from an HEV-3 strain. As shown in Fig 3D, fragment 4 of DLS13-11685 paired with a swine HEV-3 strain (AF455784) identified in Kyrgyzstan. In Fig 4C, the nucleotide sequence of ORF3 of DLS13-11685 paired with another swine HEV-3 strain (AB290312) identified in Mongolia. Interestingly, the swine HEV-3 strains identified in these two countries (AF455784 and AB290313) grouped with AB248520 (Fig 4C), the Japanese genotype 3e human HEV strain which shares a high nucleotide identity with DLS13-11685 as described above. Speculatively, the parental strains of both AB248520 and DLS13-11685 are probably related to a swine HEV-3 strain identified in Central Asia. While AB248520 identified in Japan has strictly stayed as an HEV-3 strain, the HEV-3 genome of DLS13-11685 collected in France has recombined with a rabbit HEV strain likely found in Western Europe to form a mosaic genome.

Rabbit HEV shares high amino acid sequence similarities with HEV-3: 88–91% in ORF1-encoded protein, 89–95% in ORF2-encoded protein, and 82–88% in ORF3-encoded protein. However, as examined the amino acid sequences of the three ORF-encoded proteins (S4–S6 Tables, and Tables 2 and 3), strain DLS13-11685 in fact was observed to have five amino acid residues which were present exclusively in rabbit HEV sequences but not in HEV-3, while in the same positions the two newly identified genotype 3i strains (DLS13-11677 and DLS13-11681) conversely carried the amino acid residues which were found solely in HEV-3 sequences but not in rabbit HEV, thus suggesting that DLS13-11685 may likely carry some rabbit HEV sequences, and confirming that DLS13-11677 and DLS13-11681 are HEV-3 strains. In terms of the biochemical properties, the amino acid residues in three of the five positions [aa 154 (“S”/”T”) and aa 1502 (“E”/”D”) in ORF1, and aa 241 (“I”/”L”) in ORF2] are very similar, whereas the amino acids in the remaining two positions [aa 515 (“F”/”L”/”S”) and aa 609 (“T”/”A”) in ORF1] are different, indicating that the biochemical properties do not appear to play a decisive role in selecting amino acids for the HEV strains to carry. In addition, 19 amino acid residues in ORF1-encoded protein (Table 2) and 5 in ORF2-encoded protein (Table 3) of DLS13-11685 were found either exclusively or predominantly present in rabbit HEV sequences but not or sparsely in HEV-3, whereas in the same positions the two genotype 3i strains reversely carried the amino acid residues which were either only or mainly observed in HEV-3 sequences but not or seldom in rabbit HEV. In biochemical properties, 12 of the 19 amino acids in ORF1-encoded protein (Table 2) are similar [aa 62 (“L”/”F”), aa 189 (“A”/”V”), aa 248 (“D”/”E”), aa 307 (“S”/”C”), aa 517 (“V”/”I”), aa 576 (“V”/”F”), aa 622 (“S”/”T”), aa 719 (“A”/”V”), aa 1157 (“I”/”V”), aa 1540 (“V”/”I”), aa 1594 (“I”/”V”), and aa 1645 (“I”/”V”)], but seven of them are different [aa 41 (“V”/”T”), aa 152 (“R”/”G’), aa 184 (“T”/”A”), aa 235 (“I”/”T”), aa 892 (“Q”/”H”), aa 1011 (“A”/”S”), and aa 1506 (“A”/”S”)]. Similarly, 4 of the 5 amino acids in ORF2-encoded protein (Table 3) are similar [aa 147 (“S”/”T”), aa 208 (“I”/”V”), aa 396 (“L”/”V”), and aa 645 (“I”/”V”)], but one of them is different [aa 359 (“T”/”M”)].

Of the total 29 rabbit HEV-specific amino acid residues found in DLS13-11685, 8 were in fragment 1, 15 in fragment 2, 0 in fragment 3, 1 (outside of ORF3) in fragment 4, and 5 in fragment 5. Both fragments 1 (854 nt) and 5 (1,509 nt) were designated as unclassified containing a mixture of rabbit HEV and HEV-3 sequences, and expectedly have some rabbit HEV-specific amino acid residues showing up. Fragment 3 (also unclassified, 363 nt) may have been too short to have any rabbit HEV-specific amino acid residues standing out likely due to high amino acid sequence similarities (82–95%) between rabbit HEV and HEV-3, whereas ORF3 (HEV-3, 372 nt) in fragment 4 (HEV-3, 427 nt) as expected does not have any rabbit HEV-specific amino acid residues. Fragment 2 (3,903 nt) was classified as HEV-3 derived, but harbors 15 rabbit HEV-specific amino acid residues. Taken together, it is quite possible that the parental HEV-3 sequence of DLS13-11685 has been in fact interspersed with segments of rabbit HEV sequences along the genome other than the ORF2/ORF3 overlapping region. Some regions such as fragments 1, 3 and 5 carry longer segments of rabbit HEV sequences which showed up along with HEV-3 in BootScans (Fig 2). In phylogenetic trees, all three fragments were then observed standing in between the clusters of rabbit HEV and HEV-3 (Fig 3A, 3C and 3E), and hence were designated as unclassified. Some regions such as fragments 2 and 4 obtain shorter pieces of rabbit HEV sequences which only showed up as small peaks in BootScans (Fig 2) likely due to high nucleotide sequence identities (76–79%) between rabbit HEV and HEV-3. As a result, these two fragments were found inside the cluster of HEV-3 strains in the phylogenetic trees (Fig 3B and 3D), and hence were classified as HEV-3. In other words, DLS13-11685 is a putative recombinant carrying some rabbit HEV sequences in its parental HEV-3 genome.

Rabbit HEV strains have been found in farmed, wild, pet and laboratory rabbits [20, 33], and rabbit HEV infections in humans have been confirmed recently in France [35], thus providing direct evidence of zoonotic transmission of rabbit HEV to humans. A dual infection of a patient by HEV from two distinct genotypes (HEV-3 and HEV-4) has also been documented [51]. As discussed earlier, the parental HEV-3 strain of DLS13-11685 is probably related to a swine HEV-3 strain which is also zoonotic. This can be explained by a dual infection of a swine-derived HEV-3 strain and a rabbit HEV-derived strain, resulting in the formation of an HEV-3/rabbit HEV recombinant.

In conclusion, although we have not been able to locate a segment of the DLS13-11685 genome which directly groups with any of rabbit HEV strains to provide a direct evidence that this strain is indeed a recombinant of HEV-3 and rabbit HEV strains, the results obtained by SimPlots, BootScans, phylogenetic analyses, and amino acid sequence comparisons in this study all together appear to suggest that DLS13-11685 is likely carrying some rabbit HEV-derived sequences in its parental HEV-3 genome. As a result, this newly identified strain DLS13-11685 is designated as a putative genotype 3/rabbit HEV recombinant.

## Supporting information

S1 Table Primers used to amplify and sequence the gaps of the three novel HEV genomes. (DOCX)

S2 Table Subtypes, GenBank accession numbers, and strains of 81 HEV reference sequences used in the construction of the phylogenetic trees of Figs 1, 3A–3E, 4A–4C, and S1 Fig. (DOCX)

S3 Table GenBank accession numbers of 70 HEV reference sequences along with the 81 HEV reference sequences listed in S2 Table used in the construction of the phylogenetic tree of S1 Fig. (DOCX)

S4 Table Alignment of the amino acid sequences of the ORF1-encoded proteins of 22 HEV-3 strains, 22 rabbit HEV strains, and three novel strains (DLS13-11677, DLS13-11681 and DLS13-11685). (DOCX)

S5 Table Alignment of the amino acid sequences of the ORF2-encoded proteins of 22 HEV-3 strains, 22 rabbit HEV strains, and three novel strains (DLS13-11677, DLS13-11681 and DLS13-11685). (DOCX)

S6 Table Alignment of the amino acid sequences of the ORF3-encoded proteins of 22 HEV-3 strains, 22 rabbit HEV strains, and three novel strains (DLS13-11677, DLS13-11681 and DLS13-11685). (DOCX)

S1 Fig Phylogenetic tree of strains DLS13-11677, DLS13-11681 and DLS13-11685. (DOCX)
