# Supplementary material for: Identification of a putative novel genotype 3/rabbit hepatitis E virus (HEV) recombinant
Source: PLoS One. 2018 Sep 11;13(9):e0203618. doi: 10.1371/journal.pone.0203618 (PMC6133284; doi:10.1371/journal.pone.0203618)
Supplement: S1 Table — (DOCX) [file pone.0203618.s001.docx]

**S1 Table. Primers used to amplify and sequence the gaps of the three novel HEV genomes.**

Strain DLS13-11677

| Amplicon Size (bp) | Nucleotide Position* | Primer | Sequence (5’ → 3’) |
| --- | --- | --- | --- |
| 460 | 3883 - 3900 | Sense | GCTCACGGTGTCTGATAG |
|  | 4325 - 4342 | Antisense | GAGCAGGGCTAGTATTTC |
| 685 | 4520 - 4537 | Sense | CTAATCCGGTTGTACCAT |
|  | 5187 - 5204 | Antisense | GAGGAACAGCAACAGAAC |
| 531 | 5082 - 5098 | Sense | AGACTGTTAAACCTGTG |
|  | 5596 - 5612 | Antisense | AACTGATGATGTGAGTG |

*Nucleotide position refers to the sequence of DLS13-11677 (MG783569).

Strain DLS13-11681

| Amplicon Size (bp) | Nucleotide Position* | Primer | Sequence (5’ → 3’) |
| --- | --- | --- | --- |
| 449 | 3776 - 3792 | Sense | GTGCTTACCATCAGTTA |
|  | 4207 - 4224 | Antisense | GGTGAACTTGTTACAATC |
| 468 | 4663 - 4679 | Sense | CATTGCTATGAATTTCG |
|  | 5114 - 5130 | Antisense | CCGCTGTATAATAGAAT |
| 445 | 5923 - 5939 | Sense | CCTTCACTACCGTAATC |
|  | 6351 - 6367 | Antisense | GGAGTAGAACAACTGTC |

*Nucleotide position refers to the sequence of DLS13-11681 (MG783570).

Strain DLS13-11685

| Amplicon Size (bp) | Nucleotide Position* | Primer | Sequence (5’ → 3’) |
| --- | --- | --- | --- |
| 550 | 28 - 46 | Sense | GCTCCTGGCATTACTACTG |
|  | 561 - 577 | Antisense | GATGAAGAACCACATAG |
| 259 | 492 - 509 | Sense | CTATTCACTGCACGACTT |
|  | 733 - 750 | Antisense | GTGTTCGCCGACTATCTT |
| 126 | 725 - 742 | Sense | GTACTACCAAGATAGTCG |
|  | 833 - 850 | Antisense | GAGGATATGGGACATAAG |
| 940 | 1210 - 1227 | Sense | GCCCAGAAATTTATTACG |
|  | 2132 - 2149 | Antisense | GGGAGAAATCACTAGAAA |
| 364 | 2092 - 2109 | Sense | GAGAGCACTCTGTACACT |
|  | 2438 - 2455 | Antisense | GATGGCCAGGATTAGATG |
| 225 | 2519 - 2536 | Sense | AGTTTATCATGCGTGAAG |
|  | 2726 - 2743 | Antisense | GACGATGATTACGTTCCC |
| 849 | 2723 - 2740 | Sense | CCTGGGAACGTAATCATC |
|  | 3554 - 3571 | Antisense | AAATCACACACTTCTCAG |
| 545 | 3548 - 3566 | Sense | GCCATACTGAGAAGTGT |
|  | 4076 - 4092 | Antisense | CTCCACCATGGCCTCAA |
| 440 | 4138 - 4160 | Sense | CGTGATGTATCGCGTATTACATT |
|  | 4558 - 4577 | Antisense | TCACCAGAAAGCTTCTTCCA |
| 516 | 4490 - 4507 | Sense | TTGGACTCTACCATTTAG |
|  | 4988 - 5005 | Antisense | CAATTAGGTTATGCACCA |
| 784 | 4986 - 5003 | Sense | GTTGGTGCATAACCTAAT |
|  | 5752 – 5769 | Antisense | GAAATGGAAACCGCGTAA |
| 644 | 5752 – 5769 | Sense | TTACGCGGTTTCCATTTC |
|  | 6378 - 6395 | Antisense | CGTTCTCAACAGATGTAT |
| 364 | 6290 - 6308 | Sense | CCGACAGAATTGATTTCGT |
|  | 6636 - 6653 | Antisense | TAGCCACATTGACAAATG |
| 322 | 6564 - 6581 | Sense | TCACCGGTGCTAACTATG |
|  | 6868 - 6885 | Antisense | GAGATAGCAACAAGGTAG |
| 259 | 6866 - 6883 | Sense | GGCTACCTTGTTGCTATC |
|  | 7108 – 7124 | Antisense | ATTAAGACTCCCGGGTT |

*Nucleotide position refers to the sequence of DLS13-11685 (MG783571).
