# Supplementary material for: Identification of a putative novel genotype 3/rabbit hepatitis E virus (HEV) recombinant
Source: PLoS One. 2018 Sep 11;13(9):e0203618. doi: 10.1371/journal.pone.0203618 (PMC6133284; doi:10.1371/journal.pone.0203618)
Supplement: S2 Table — (DOCX) [file pone.0203618.s002.docx]

**S2 Table. Subtypes, GenBank accession numbers, and strains of 81 HEV reference sequences used in the construction of the phylogenetic trees of Figs 1, 3A-E, 4A-C, and S1 Fig.**

| Genotype | Subtype-GenBank accession # (Strain) |
| --- | --- |
| HEV-1 (11) | 1a-M73218 (Burma), 1b-D11092 (HPECG), 1c-X98292 (I1), 1d-AY230202 (Morocco), 1e-AY204877 (T3), 1f-JF443721 (IND-HEV-AVH5-2010), 1a-AF051830 (TK15/92), 1-D11093 (Uigh179), 1-AF459438 (Yam67), 1-M80581 (Sar55), 1-AF185822 (Abb-2B) |
| HEV-2 (1) | 2a-M74506 (M1) |
| HEV-3 (22) | 3a-AF082843 (Swine HEV), 3b-AP003430 (JRA1), 3i-FJ705359 (wbGER27), 3e-AB248521 (swJ8-5), 3f-AB369687 (E116-YKH98C), 3g-AF455784 (Osh-205), 3h-JQ013794 (TR19), 3i-FJ998008 (BB02), 3j-AY115488 (swArkell), 3-AB290312 (swMN06-A1288), 3h-JQ953664 (FR-SHEV-3c-like), 3x-AB369689 (E088STM04C), 3h-AB290313 (swMN06-C1056), 3-EU360977 (swX07-E1), 3-EU723513 (SW627), 3-AF060668 (US1), 3-AB073912 (swJ570), 3-AB189070 (JBOAR-1Hyo04), 3i-KU176129 (TLS-B), 3i-KJ701409 (AB_7110), 3e-AB248520 (HE-JA04-1911), 3b-AB291962 (JHK-Toy04C) |
| HEV-3 (Rabbit HEV)  (22) | 3ra-FJ906895 (GDC9, China), 3-FJ906896 (GDC46, China), 3-GU937805 (ch-bj-n1, China), 3-JQ013791 (W1-11, France), 3-JQ013792 (W7-57, France), 3-JQ013793 (TLS-18516, France), 3-KJ013414 (CHN-BJ-r14(8), China), 3-KJ013415 (CHN-BJ-r14(9), China), 3-JX109834 (CHN-BJ-R14, China), 3-JX121233 (CHN-BJ-R14, China), 3-JQ768461 (CHN-BJ-rb14, China), 3-JX565469 (CMC-1, USA), 3-AB740220 (rblM199, China), 3-AB740221 (rblM022, China), 3-AB740222 (rblM004, China), 3-KX227751 (CHN-SX-rHEV, China), 3-KY496200 (KOR-Rb-1, Korea), 3-KY436898 (ME-2016-rab52, Germany), 3-MF480297 (R42, Germany), 3-MF480298 (U46, Germany), 3rb-MG211750 (8574/13s, stool, France), 3rb-MG211751 (1, serum, France) |
| HEV-4 (17) | 4h-GU119961 (CHN-XJ-SW13), 4-GU206559 (bjsw1), 4-AB097812 (HE-JA1), 4f-AB220974 (HE-JA2), 4a-AB197673 (JKO-ChiSai98C), 4e-AY723745 (IND-SW-00-01), 4d-AJ272108 (T1), 4b-DQ279091 (swDQ), 4g-AB108537 (CCC220), 4i-DQ450072 (swCH31), 4c-AB074915 (JAK-Sai), 4-AB369688 (E087-SAP04C), 4-EU676172 (swGX40), 4-EU366959 (swGX32), 4-AY594199 (swCH25), 4-GU361892 (hb-3), 4-GU119960 (CHN-XJ-SW33) |
| HEV-5  (Wild boar HEV)  (1) | 5a-AB573435 (JBOAR135_Shiz09) |
| HEV-6  (Wild boar HEV)  (2) | 6a-AB602441 (wbJOY_06), 6-AB856243 (wbJNN_13) |
| HEV-7  (Camel HEV)  (2) | 7a-KJ496143 (178C), 7-KJ496144 (180C) |
| HEV-8  (Camel HEV)  (3) | 8-KX387865 (BcHEV-12XJ), 8-KX387866 (BcHEV-48XJ),  8-KX387867 (BcHEV-62XJ) |
