# Supplementary material for: Identification of a putative novel genotype 3/rabbit hepatitis E virus (HEV) recombinant
Source: PLoS One. 2018 Sep 11;13(9):e0203618. doi: 10.1371/journal.pone.0203618 (PMC6133284; doi:10.1371/journal.pone.0203618)
Supplement: S3 Table — (DOCX) [file pone.0203618.s003.docx]

**S3 Table. GenBank accession numbers of 70 HEV reference sequences along with the 81 HEV reference sequences from S2 Table used in the construction of the phylogenetic tree of S1 Fig.**

| Genotype | GenBank Accession # |
| --- | --- |
| HEV1 (5) | D10330, FJ457024, L08816, L25595, X99441 |
| HEV3 (58) | AB074918, AB089824, AB091394, AB189071, AB189074, AB222182, AB222183, AB222184, AB236320, AB248522, AB291955, AB291958, AB291961, AB362842, AB362843, AB425830, AB443623, AB443625, AB481226, AB481228, AB481229, AB630970, AB630971, AB740232, AB850879, AF060669, AY575857, AY575858, AY575859, EU375463, EU495148, EU723512, EU723514, EU723515, EU723516,  FJ426403, FJ426404, FJ527832, FJ653660, HM055578,  JN906976, KC166967, KC166968, KC166969, KC166970, KC618402, KP294371, KT159771, KT447526, KT447528, KU176130, KU176131, KU176132, KX462160, KY780957, LC131066, MF346772, MF346773 |
| HEV4 (5) | AB091395, AB161717, AB220971, AB291959, FJ610232 |
| Rat HEV (2) | GU345042, GU345043 |
