# Supplementary material for: Identification of a putative novel genotype 3/rabbit hepatitis E virus (HEV) recombinant
Source: PLoS One. 2018 Sep 11;13(9):e0203618. doi: 10.1371/journal.pone.0203618 (PMC6133284; doi:10.1371/journal.pone.0203618)
Supplement: S4 Table — (DOCX) [file pone.0203618.s004.docx]

**S4 Table.** Alignment of the amino acid sequences of the ORF1-encoded proteins of 22 HEV-3 strains, 22 rabbit HEV strains, and three novel strains (DLS13-11677, DLS13-11681 and DLS13-11685).

38 nt (aa 11)

Majority MEAHQFIKAPGITTAIEQAALAAANSALANAVVVRPFLSRTQTEILINLMQPRQLVFRPEVFWNHPIQRVIHNELEQYCR

---------+---------+---------+---------+---------+---------+---------+---------+

10 20 30 40 50 60 70 80

---------+---------+---------+---------+---------+---------+---------+---------+

DLS11685_ORF1.pro ...............L............................V..........I........................

KJ701409_ORF1.pro ..................X.....................V....................L..................

KU176129_ORF1.pro ........................................V....................L..................

AB248520_ORF1.pro ...........................................D.................L..................

AB291962_ORF1.pro ........................................V....................L..................

DLS11677_ORF1.pro ........................................V....................L..................

DLS11681_ORF1.pro ........................................V....................L..................

JQ953664_ORF1.pro ........................................V....................L..................

AB073912_ORF1.pro ........................................V....................L..................

AB189070_ORF1.pro ........................................V....................L..................

AB248521_ORF1.pro ...........................................D.................L..................

AB290312_ORF1.pro ........................................V....................L..................

AB290313_ORF1.pro ........................................G..D.................L..................

AB369687_ORF1.pro .............................................................L..................

AB369689_ORF1.pro ........................................V.......................................

AF060668_ORF1.pro ---------...............................V....................L..................

AF082843_ORF1.pro ........................................V....................L.......A..........

AF455784_ORF1.pro ........................................V....................L..................

AP003430_ORF1.pro ........................................V....................L..................

AY115488_ORF1.pro ........................................V....................L..................

EU360977_ORF1.pro ........................................V....................L..................

EU723513_ORF1.pro ........................................V..D....................................

FJ705359_ORF1.pro ........................................V....................L..................

FJ998008_ORF1.pro ........................................V....................L..................

JQ013794_ORF1.pro ------------............................L....................L..................

AB740220_ORF1.pro .................................I..............................................

AB740221_ORF1.pro .................................I..............................................

AB740222_ORF1.pro .................................I.........D....................................

FJ906895_ORF1.pro .................................I.........D....................................

FJ906896_ORF1.pro ...RR......LP..L..F....G......G..I.........D......P....G...S....................

GU937805_ORF1.pro ...............................E.I..............................................

JQ013791_ORF1.pro ...........V...................................................S................

JQ013792_ORF1.pro ...........................................D...................S................

JQ013793_ORF1.pro -................................I..............................................

JQ768461_ORF1.pro .................................I..............................................

JX109834_ORF1.pro .................................I..............................................

JX121233_ORF1.pro .................................I..............................................

JX565469_ORF1.pro .................................I..............................................

KJ013414_ORF1.pro .................................I..............................................

KJ013415_ORF1.pro .................................I..............................................

KX227751_ORF1.pro .................................I..............................................

KY436898_ORF1.pro .................................I..............................................

KY496200_ORF1.pro .................................I......L.......................................

MF480297_ORF1.pro ................................................................................

MF480298_ORF1.pro .................................I..............................................

MG211750_ORF1.pro .................................I......L.......................................

MG211751_ORF1.pro .................................I......V.......................................

Majority ARAGRCLEVGAHPRSINDNPNVLHRCFLRPVGRDVQRWYSAPTRGPAANCRRSALRGLPPVDRTYCFDGFSRCAFAAETG

---------+---------+---------+---------+---------+---------+---------+---------+

90 100 110 120 130 140 150 160

---------+---------+---------+---------+---------+---------+---------+---------+

DLS11685_ORF1.pro ............................................................S..........G.T......

KJ701409_ORF1.pro .........................................................................S......

KU176129_ORF1.pro ...............V.........................................................S......

AB248520_ORF1.pro ................................................................................

AB291962_ORF1.pro ........I.......................................................................

DLS11677_ORF1.pro .........................................................................S......

DLS11681_ORF1.pro .........................................................................S......

JQ953664_ORF1.pro ............................................................A...................

AB073912_ORF1.pro ................................................................................

AB189070_ORF1.pro ........I...................................................A...................

AB248521_ORF1.pro ................................................................................

AB290312_ORF1.pro ................................................................................

AB290313_ORF1.pro .............................................................................G..

AB369687_ORF1.pro ................................................................................

AB369689_ORF1.pro S........................................................................S......

AF060668_ORF1.pro ............................................................A...................

AF082843_ORF1.pro ....C.........F.................................................................

AF455784_ORF1.pro ................................................................................

AP003430_ORF1.pro ........I..............................................................S........

AY115488_ORF1.pro ............................K................................................K..

EU360977_ORF1.pro ................................................................................

EU723513_ORF1.pro ................................................................................

FJ705359_ORF1.pro .........................................................................S......

FJ998008_ORF1.pro ................................................................................

JQ013794_ORF1.pro ........I.......................................................................

AB740220_ORF1.pro .....................I......................................T..........S.M......

AB740221_ORF1.pro ............................................................A..........G.T......

AB740222_ORF1.pro .....................I.................................................S.T......

FJ906895_ORF1.pro .....................I.................................................S.T......

FJ906896_ORF1.pro ............................................................A..........G.P......

GU937805_ORF1.pro ............................................................A..........G.T......

JQ013791_ORF1.pro ..........................................A...........................A.........

JQ013792_ORF1.pro ..........................................A.................A.........AG........

JQ013793_ORF1.pro .......................................................................G........

JQ768461_ORF1.pro ............................................................A..........G.T......

JX109834_ORF1.pro ............................................................A..........G.T......

JX121233_ORF1.pro ............................................................A..........G.T......

JX565469_ORF1.pro S..................S.I.................................................S.T......

KJ013414_ORF1.pro ............................................................A..........G.T......

KJ013415_ORF1.pro ............................................................A..........G.T......

KX227751_ORF1.pro .....................I.................................................G.M......

KY436898_ORF1.pro .....................I......................................A..........G.T......

KY496200_ORF1.pro ......................................................................AG.T......

MF480297_ORF1.pro .....................I..............................T..................G........

MF480298_ORF1.pro .....................I........A........................................G........

MG211750_ORF1.pro .......................................T...................A...........A........

MG211751_ORF1.pro .......................................T...................A...........A........

Majority IALYSLHDLWPADVAEAMARHGMTRLYAVLHLPPEVLLPPGTYHTTSYLLIHDGDRAVVTYEGDTSAGYNHDVSILRAWI

---------+---------+---------+---------+---------+---------+---------+---------+

170 180 190 200 210 220 230 240

---------+---------+---------+---------+---------+---------+---------+---------+

DLS11685_ORF1.pro L......................A...V..........................K...................T.....

KJ701409_ORF1.pro V...........................A...................................................

KU176129_ORF1.pro V...........................A...................................................

AB248520_ORF1.pro ............................A...................................................

AB291962_ORF1.pro V...........................A...................................................

DLS11677_ORF1.pro V...........................A...................................................

DLS11681_ORF1.pro V...........................A...................................................

JQ953664_ORF1.pro V...........................A.........................N.........................

AB073912_ORF1.pro V...........................A...................................................

AB189070_ORF1.pro V...........................A.........................N.........................

AB248521_ORF1.pro ............................A...................................................

AB290312_ORF1.pro V...........................A................S..................................

AB290313_ORF1.pro ...........TV...................................................................

AB369687_ORF1.pro ...........S................A.........................N.........................

AB369689_ORF1.pro V...........................A.........................N.........................

AF060668_ORF1.pro V.....................X.....A...................................................

AF082843_ORF1.pro V...........................A...L...............................................

AF455784_ORF1.pro ............................A...................................................

AP003430_ORF1.pro V...........................A.........................N.........................

AY115488_ORF1.pro V...........................A.........................S.........................

EU360977_ORF1.pro ...........S....................................................................

EU723513_ORF1.pro ...........S..........................................N.........................

FJ705359_ORF1.pro V...........................A...................................................

FJ998008_ORF1.pro V...........................A...................................................

JQ013794_ORF1.pro V...........................A...................................................

AB740220_ORF1.pro ...................H...S..............................S.........................

AB740221_ORF1.pro ...................H..LA..............................A.........................

AB740222_ORF1.pro ...................H...S..............................S.........................

FJ906895_ORF1.pro V..................H...S..............................S.........................

FJ906896_ORF1.pro ...................H..LA..............................S.........................

GU937805_ORF1.pro ...................H..PA................SA............A.........................

JQ013791_ORF1.pro V..................H.........................A........N...I...............T.....

JQ013792_ORF1.pro V..................H.........................A........N...................T.....

JQ013793_ORF1.pro ...................H..................................S...................T.....

JQ768461_ORF1.pro ...................H..LA......................T.......A.........................

JX109834_ORF1.pro ...................H..LA......................T.......A.........................

JX121233_ORF1.pro ...................H..LA......................T.......A.........................

JX565469_ORF1.pro F..D...............H...A..............................S.........................

KJ013414_ORF1.pro ...................H..LA...........A..........T.......A.........................

KJ013415_ORF1.pro ...................H..LA...........A..........T.......A.........................

KX227751_ORF1.pro ...................H...S..............................N.........................

KY436898_ORF1.pro ...................H..................................S...................V.....

KY496200_ORF1.pro ...................H..................................S.........................

MF480297_ORF1.pro ........M..........................................N..S.V.......................

MF480298_ORF1.pro ........M.............................................S...................T.....

MG211750_ORF1.pro ............................A.........................S...................T.....

MG211751_ORF1.pro ............................A.........................S...................T.....

891 nt (aa 296)

Majority RTTKIVGDHPLVIERVRAIGCHFVLLLTAAPEPSPMPYVPYPRSTEVYVRSIFGPGGSPSLFPSACSTKSTFHAVPVHIW

---------+---------+---------+---------+---------+---------+---------+---------+

250 260 270 280 290 300 310 320

---------+---------+---------+---------+---------+---------+---------+---------+

DLS11685_ORF1.pro .......E..........................................................C.............

KJ701409_ORF1.pro ................................................................................

KU176129_ORF1.pro ................................................................................

AB248520_ORF1.pro ................................................................................

AB291962_ORF1.pro ................................................................................

DLS11677_ORF1.pro ....................S...........................................................

DLS11681_ORF1.pro ................................................................................

JQ953664_ORF1.pro ................................................................................

AB073912_ORF1.pro ................................................................................

AB189070_ORF1.pro ................................................................................

AB248521_ORF1.pro ................................................................................

AB290312_ORF1.pro .............................................................L..................

AB290313_ORF1.pro ................................................................................

AB369687_ORF1.pro ................................................................................

AB369689_ORF1.pro ................................................................................

AF060668_ORF1.pro ................................................................................

AF082843_ORF1.pro ................................................................................

AF455784_ORF1.pro ................................................................................

AP003430_ORF1.pro ................................................................................

AY115488_ORF1.pro ................................................................................

EU360977_ORF1.pro ................................................................................

EU723513_ORF1.pro ................................................................................

FJ705359_ORF1.pro ................................................................................

FJ998008_ORF1.pro ................................................................................

JQ013794_ORF1.pro ................................................................................

AB740220_ORF1.pro .......E................................F......F..................C.............

AB740221_ORF1.pro ....VI..................................F......F................................

AB740222_ORF1.pro .....I.E................................F......F..................C.....Y.......

FJ906895_ORF1.pro .....I.E................................F......F..................C.....Y.......

FJ906896_ORF1.pro ....VT...............................-AGRRF....F............P...................

GU937805_ORF1.pro ....VT..................................F......F................................

JQ013791_ORF1.pro ................................................................................

JQ013792_ORF1.pro ................................................................................

JQ013793_ORF1.pro ........................................F...................................A...

JQ768461_ORF1.pro ....VT..................................F......F......................I.........

JX109834_ORF1.pro ....VT..................................F......F......................I.........

JX121233_ORF1.pro ....VT..................................F......F................................

JX565469_ORF1.pro .....T..................................F......F................................

KJ013414_ORF1.pro ....VT..................................F...A..F......................I.........

KJ013415_ORF1.pro ....VT..................................F...A..F......................I.........

KX227751_ORF1.pro .....I.E.......................................F..................C.............

KY436898_ORF1.pro ....VI..................................F......F................................

KY496200_ORF1.pro .....T..................................F......F................................

MF480297_ORF1.pro .......E................................F......F................................

MF480298_ORF1.pro .....I..................................F......F................................

MG211750_ORF1.pro ....X..........................................F................................

MG211751_ORF1.pro ....X..........................................F................................

Majority DRLMLFGATLDDQAFCCSRLMTYLRGISYKVTVGALVANEGWNASEDALTAVITAAYLTICHQRYLRTQAISKGMRRLEV

---------+---------+---------+---------+---------+---------+---------+---------+

330 340 350 360 370 380 390 400

---------+---------+---------+---------+---------+---------+---------+---------+

DLS11685_ORF1.pro ........................................................................R.......

KJ701409_ORF1.pro ................................................................................

KU176129_ORF1.pro ................................................................................

AB248520_ORF1.pro ................................................................................

AB291962_ORF1.pro ................................................................................

DLS11677_ORF1.pro ................................................................................

DLS11681_ORF1.pro ................................................................................

JQ953664_ORF1.pro ................................................................................

AB073912_ORF1.pro ................................................................................

AB189070_ORF1.pro ................................................................................

AB248521_ORF1.pro ..............................X.................................................

AB290312_ORF1.pro ................................................................................

AB290313_ORF1.pro ................................................................................

AB369687_ORF1.pro ................................................................................

AB369689_ORF1.pro ................................................................................

AF060668_ORF1.pro ...................................................X..........................G.

AF082843_ORF1.pro ................................................................................

AF455784_ORF1.pro ................................................................................

AP003430_ORF1.pro ................................................................................

AY115488_ORF1.pro ................................................................................

EU360977_ORF1.pro ................................................................................

EU723513_ORF1.pro ...............................................................................I

FJ705359_ORF1.pro ................................................................................

FJ998008_ORF1.pro ................................................................................

JQ013794_ORF1.pro ................................................................................

AB740220_ORF1.pro ..................................T.............................................

AB740221_ORF1.pro ...............................................................................L

AB740222_ORF1.pro ..................................T.............................................

FJ906895_ORF1.pro ..................................T.............................................

FJ906896_ORF1.pro ...............................................................................L

GU937805_ORF1.pro ............................H..................................................L

JQ013791_ORF1.pro ..................................T............................................L

JQ013792_ORF1.pro ...............................................................................L

JQ013793_ORF1.pro ................................................................................

JQ768461_ORF1.pro ...............................................................................L

JX109834_ORF1.pro ...............................................................................L

JX121233_ORF1.pro ...............................................................................L

JX565469_ORF1.pro .........................................................P......................

KJ013414_ORF1.pro .......................................................................L.......L

KJ013415_ORF1.pro .......................................................................L.......L

KX227751_ORF1.pro ..................................T.............................................

KY436898_ORF1.pro ...............................................................................L

KY496200_ORF1.pro ...............................................................................L

MF480297_ORF1.pro ...................................................................I............

MF480298_ORF1.pro ..................................T.............................................

MG211750_ORF1.pro ..................................T............................................L

MG211751_ORF1.pro ..................................T............................................L

Majority EHAQKFITRLYSWLFEKSGRDYIPGRQLQFYAQCRRWLSAGFHLDPRVLVFDESVPCRCRSFLKKVAGKFCCFMKWLGQE

---------+---------+---------+---------+---------+---------+---------+---------+

410 420 430 440 450 460 470 480

---------+---------+---------+---------+---------+---------+---------+---------+

DLS11685_ORF1.pro ............................................................A..GRAV.RL..........

KJ701409_ORF1.pro ......................................................A.....T...................

KU176129_ORF1.pro ............................................................T....AV.............

AB248520_ORF1.pro .....................................................A..........................

AB291962_ORF1.pro ............................................................T...R.........R.....

DLS11677_ORF1.pro ......................................................A.....T...................

DLS11681_ORF1.pro .......I..............................................A.....T....A........R.....

JQ953664_ORF1.pro ............................................................T...................

AB073912_ORF1.pro ............................................................T.............R.....

AB189070_ORF1.pro ............................................................T.............R.....

AB248521_ORF1.pro .....................................................A..........................

AB290312_ORF1.pro ............................................................T...................

AB290313_ORF1.pro .....................................................A...............S..........

AB369687_ORF1.pro .....................................................A..........................

AB369689_ORF1.pro ............................................................T....A.S......R.....

AF060668_ORF1.pro ............................................................T.............R.....

AF082843_ORF1.pro ............................................................T.............R.....

AF455784_ORF1.pro .................................................................A...I..........

AP003430_ORF1.pro ............................................................T.............R.....

AY115488_ORF1.pro ............................................................T.I.................

EU360977_ORF1.pro .....................................................A..........................

EU723513_ORF1.pro .....................................................A..........................

FJ705359_ORF1.pro ............................................................T....AV.............

FJ998008_ORF1.pro ............................................................T...................

JQ013794_ORF1.pro ............................................................T...................

AB740220_ORF1.pro .......................................................H.....LIRN..R.V..........

AB740221_ORF1.pro .......................................................H......V.N..R.V..........

AB740222_ORF1.pro ......V................................................H.....LIRS.TR.V..........

FJ906895_ORF1.pro ......V................................................H.....LVRSA.R.V..........

FJ906896_ORF1.pro .......................................................H......V.N..T.I........R.

GU937805_ORF1.pro ............................E..........................H......V.N..R.V..........

JQ013791_ORF1.pro ..S....................................................H....GLFRS..R.A..........

JQ013792_ORF1.pro ..S....S...............................................H.....L..N..R.A..........

JQ013793_ORF1.pro .......................................................H......V.N.TR.I..........

JQ768461_ORF1.pro ............................E..........................H......V.N..R.V..........

JX109834_ORF1.pro ............................E..........................H......V.N..R.V..........

JX121233_ORF1.pro ............................E..........................H......V.N..R.V..........

JX565469_ORF1.pro .......................................................H......V.N..R.V..........

KJ013414_ORF1.pro ............................E..........................H......V.N..R.V..........

KJ013415_ORF1.pro ............................E..........................H......V.N..R.V..........

KX227751_ORF1.pro .......................................................H.....LIRN.TR.V..........

KY436898_ORF1.pro .......................................................H......V.S.TK.V..........

KY496200_ORF1.pro .......................................................H....G.V.N..R.V..........

MF480297_ORF1.pro ......V.............N......I...........................H....N.V.N..A.A....R.....

MF480298_ORF1.pro ....................N....H.I...........................H......VRS.TK.V....R.....

MG211750_ORF1.pro ...............................................L.......H......I.N.TR.V..........

MG211751_ORF1.pro ...............................................L.......H......I.N.TR.V..........

Majority CTCFLEPAEGLVGDQGHDNEAYEGSEVDPAEPAHLDVSGTYAVHGRQLEALYRALNIPHDIAARAARLTATVELVAGPGR

---------+---------+---------+---------+---------+---------+---------+---------+

490 500 510 520 530 540 550 560

---------+---------+---------+---------+---------+---------+---------+---------+

DLS11685_ORF1.pro ..............C...................S.I......Y.H......G.....RE..T..SH.A....VS.S...

KJ701409_ORF1.pro ..............H.............Q............T.......................S........T...D.

KU176129_ORF1.pro ..............H.............Q....................................S............D.

AB248520_ORF1.pro ...........T..C.............S....................................S........T.S...

AB291962_ORF1.pro .......V....................Q.....F.......................................A...D.

DLS11677_ORF1.pro ..............R.............R...V.F........Y....V................S........T...D.

DLS11681_ORF1.pro ..............H...........I.Q....................................S........A...D.

JQ953664_ORF1.pro ........D...................Q..............Y..............Q......S..........S.D.

AB073912_ORF1.pro .................................YF.......................................A.S.D.

AB189070_ORF1.pro ...........I..H.............Q....................T........Q...................D.

AB248521_ORF1.pro ...........I..C..................................................S........T.S...

AB290312_ORF1.pro ............................Q..L..I..............................S..........S.D.

AB290313_ORF1.pro ..............C.................VPV..............P...............S..........S...

AB369687_ORF1.pro ..............C...........................................D......S........A.S...

AB369689_ORF1.pro .................................................................S............D.

AF060668_ORF1.pro ..............H..............................H..........V.Q......S..........S.D.

AF082843_ORF1.pro ..............Y.........................................V........S........T.S.D.

AF455784_ORF1.pro ..............C......F....................................Q......S........A.S...

AP003430_ORF1.pro ............................Q...V............H............................A...D.

AY115488_ORF1.pro .......................D....R....Y.........Y.H...............V...S..........S.D.

EU360977_ORF1.pro ..............C.....................I........S...................S..........S...

EU723513_ORF1.pro ..............C............................Y.....................S..........S...

FJ705359_ORF1.pro ..............H.............Q................................V...S........A...D.

FJ998008_ORF1.pro ..............H.............Q...V................................S............D.

JQ013794_ORF1.pro ..............H.............Q....................................S........A...D.

AB740220_ORF1.pro ...........L.....................PT......T....L.VD..K...V.Q..V......S.....AV...Q

AB740221_ORF1.pro ...........I.....................PS......V...QL.VD......V...........S.....T.....

AB740222_ORF1.pro ...........L.....................PT...........L.VD..K...V.QE.....V..S.....A.....

FJ906895_ORF1.pro .......V...L.....................PT......T....L.VD..K...V.QE.V......P.....AV....

FJ906896_ORF1.pro ...........I.....................PS......I...QL.VD......V....V......S......T....

GU937805_ORF1.pro ...........I.....................PS..........QL.VD......V...........S.....T.....

JQ013791_ORF1.pro .S.....V...L.............D.......PT.I....V...Q..VD........R.LE......S....IT.SQ..

JQ013792_ORF1.pro .S.....V...L.............D.......PT.I....V......VD........R.LE......S....ITT..A.

JQ013793_ORF1.pro ...........L..V..................FS.I....T...QL.VD......V.D.........S.....A.S...

JQ768461_ORF1.pro ...........I.....................PS......V...QL.VD......V...........S.....T.....

JX109834_ORF1.pro ...........I.....................PS......V...QL.VD......V...........S.....T.....

JX121233_ORF1.pro ...........I.....................PS......V...QL.VD......V...........S.....T.....

JX565469_ORF1.pro ...........I.....................PS......T...QL.VD......V...........S.....A.....

KJ013414_ORF1.pro ...........I.....................PS......V...QL.VD......V...........S.....T.....

KJ013415_ORF1.pro ...........I.....................PS......V...QL.VD......V...........S.....T.....

KX227751_ORF1.pro ...........L.....................PT......T....L.VD..K...V.Q..V......S.....A.....

KY436898_ORF1.pro ...........L.....................LS.I....T...QL.AD..K.....D.V....V..S...........

KY496200_ORF1.pro ...........L..............I......PS......I...QL.VD......V.Q..V......S.....A.....

MF480297_ORF1.pro ...........L.....................CS.I....T....L.MD........P.L.......S........Q..

MF480298_ORF1.pro ...........L.....................LP.I..I.T....L.TD........P.L..Q.T..S.......SQ..

MG211750_ORF1.pro ...........I..............I....T.LP.I....V....L.AD..................S......T....

MG211751_ORF1.pro ...........I..............I....T.LP.I....V....L.AD..................S......T....

Majority LECRTVLGNKTFRTTVTDGAHLEANGPEQYVLSFDESRQCMGAGPHSLTYELTPAGLQVKISSNGLDCTAVFPPGGAPSA

---------+---------+---------+---------+---------+---------+---------+---------+

570 580 590 600 610 620 630 640

---------+---------+---------+---------+---------+---------+---------+---------+

DLS11685_ORF1.pro .D...T......S..FV...R..V...D.......D.L.S........A.....G..R.RVT......EVT.A..A....

KJ701409_ORF1.pro ................V..................A.C.S......N.......................T.L.......

KU176129_ORF1.pro ................V..................A...S........S.....................T.........

AB248520_ORF1.pro ..............SI.......V........T..D...A...................R....................

AB291962_ORF1.pro ................V..................A.S.S....S..............R....................

DLS11677_ORF1.pro ................V..................A.C.S......N.......S.....F.F..P....T.........

DLS11681_ORF1.pro ................V..................A.C.S....T.N.......................T.........

JQ953664_ORF1.pro .....I....V.....V......V...........A.H.S.................L............T.S.......

AB073912_ORF1.pro .....II.........V......V...........A...T....S..............R....................

AB189070_ORF1.pro ..........I..................C.....A...S....S.........................I.........

AB248521_ORF1.pro ..............SI.......V........T..D...A........................................

AB290312_ORF1.pro ................V...............T..A...S....S.........................T.........

AB290313_ORF1.pro ..............SI................T..D...V....S...M...V..........S......I.........

AB369687_ORF1.pro ..............SI................T..D.H.A............V.................I.........

AB369689_ORF1.pro ................V..................AT..S....S..............N..........T.........

AF060668_ORF1.pro ................V..................A...S....S..............R..........T.........

AF082843_ORF1.pro ................V..................A...S....S..............R..........T.........

AF455784_ORF1.pro ................V...............T..D.X.S...................R....................

AP003430_ORF1.pro ...................................A...S....S...................................

AY115488_ORF1.pro ..........V.....V......V.....H.....VL..S....S........S.....R..........T.S.......

EU360977_ORF1.pro ..............SIV...............T..D...AI...........V....R...T..................

EU723513_ORF1.pro ..............SIA...............T..D...A............V......R..........T.S.......

FJ705359_ORF1.pro ................V..................A...S........S.....................T........V

FJ998008_ORF1.pro ................V..................A...S......N......S.......V........T.........

JQ013794_ORF1.pro ................V..................A...S....S............L............T.........

AB740220_ORF1.pro .....I.........F....Q...............A...........A.....S......T.............K...V

AB740221_ORF1.pro .....I.........I....Q.......X.......A...........V.D...S.............L......K...V

AB740222_ORF1.pro .....I........VF....Q...............V...........V.....R......T......I......K..GV

FJ906895_ORF1.pro .....T...N....MF....Q...............A...........V.....S......T.............K...V

FJ906896_ORF1.pro .....I.........I....Q...............AH..........V.....S....RV.......M......R....

GU937805_ORF1.pro .....I.........I....Q.............G.AG..........V....SS.............V......R...V

JQ013791_ORF1.pro .....I..........A..T................A....S......A.....G....M...............R..KT

JQ013792_ORF1.pro ....................A...............A...VS......A.....G..K.M..........L....R..NV

JQ013793_ORF1.pro .D...I.........IV...Q...............TH..........A.....G..R..........V....X.R...P

JQ768461_ORF1.pro .....I.........I....Q...............AG..........V....SS.............V....S.R...V

JX109834_ORF1.pro .....I.........I....Q...............AG..........V....SS.............V....S.R...V

JX121233_ORF1.pro .....I.........I....Q...............AG..........V....SS.P...........V....S.R...V

JX565469_ORF1.pro .....I.........I....Q...............A...........V.....S.............V......R...V

KJ013414_ORF1.pro .....I.........I....Q...............AG..........V....SS.............V....S.R...V

KJ013415_ORF1.pro .....I.........I....Q...............AG..........V..F.SS.F..........RV....S.R...V

KX227751_ORF1.pro .....I.........F....Q...............A...........V.....S......T......M....S.R....

KY436898_ORF1.pro .....F.........IV...Q...............AH......Q...V.....N....................R....

KY496200_ORF1.pro .....I.........I....Q...............AH.......Y..V....SS...........E........R...V

MF480297_ORF1.pro .....I........II....Q...............A.....V.QC..A.....S..R....C.....A.I....Q..R.

MF480298_ORF1.pro .....I........V.....Q...............AH....V.QY..A.....G.......C.....V......R..GV

MG211750_ORF1.pro .....I..........V...R.........A.....AH..........A.....S..R...T......V......R...V

MG211751_ORF1.pro .....I..........V...Q.........A.....AH..........A.....S......T......V......R...V

Majority APGEVAAFCSALYRYNRFTQRHSLVGGLWLHPEGLLGLFPPFSPGHLWESANPFCGEGTLYTRTWSTSGFSSDFSPPEAA

---------+---------+---------+---------+---------+---------+---------+---------+

650 660 670 680 690 700 710 720

---------+---------+---------+---------+---------+---------+---------+---------+

DLS11685_ORF1.pro T.......................A............I........V..........S...................PV.

KJ701409_ORF1.pro D.......................T............I..........................................

KU176129_ORF1.pro ........................I............I..........................................

AB248520_ORF1.pro ........................T.....................I.................................

AB291962_ORF1.pro .................H......T............I........I.................................

DLS11677_ORF1.pro D.......................T............I..................................V.......

DLS11681_ORF1.pro D.......................T............I..........................................

JQ953664_ORF1.pro ........................A............I..........................................

AB073912_ORF1.pro ........................T............I........I.................................

AB189070_ORF1.pro ........................T............I........I.................................

AB248521_ORF1.pro ..S.....................T.....................I.................................

AB290312_ORF1.pro ........................T............I..........................................

AB290313_ORF1.pro ........................T....................................................D..

AB369687_ORF1.pro ........................T................................S......................

AB369689_ORF1.pro ........................T............I........T.................................

AF060668_ORF1.pro ........................T............I........I.................................

AF082843_ORF1.pro ........................T............I........I..P..............................

AF455784_ORF1.pro E.......................T.......................................................

AP003430_ORF1.pro P.......................T............V........I.................................

AY115488_ORF1.pro ........................T............I........I.................................

EU360977_ORF1.pro T.......................T................................S......................

EU723513_ORF1.pro S.......................T................................S......................

FJ705359_ORF1.pro ........................I............I..........................................

FJ998008_ORF1.pro ........................T............I....................................A.....

JQ013794_ORF1.pro ........................T............I..........................................

AB740220_ORF1.pro GAA..T...........TI....I......................S..........S........E.......L...V.

AB740221_ORF1.pro GAA..T...........TI....I......................F...N......E........M............T

AB740222_ORF1.pro EAA..T...........TI....V......................F...T...............E.......L....V

FJ906895_ORF1.pro EAA..T...........TI....I........G.............F...T...............E..S....L....V

FJ906896_ORF1.pro GAA..T...........TI....I......................F...T............................T

GU937805_ORF1.pro GAA..T...........TI....I......................F...N...............M............T

JQ013791_ORF1.pro E.A......A.......TI....I.............G...................S....K...........A....G

JQ013792_ORF1.pro E.A......A.......TI....V.............C...................S................A.L..G

JQ013793_ORF1.pro .AA.XT...........TI...........................F.......S..S......................

JQ768461_ORF1.pro GAA..T...........TI....I......................F...N...............M............T

JX109834_ORF1.pro GAA..T...........TI....I......................F...N...............M............T

JX121233_ORF1.pro GAA..T...........TI....I......................F...N...............M............T

JX565469_ORF1.pro GAA..T...........TI....I......................F...T............................T

KJ013414_ORF1.pro GAA..T...........TI....I......................F...N...............M............T

KJ013415_ORF1.pro GAA..T...........TI....I......................F...N...............M............T

KX227751_ORF1.pro EAA..T...........TI....I......................F...T...............E...........V.

KY436898_ORF1.pro GAA..S...........AI......................................E....K.................

KY496200_ORF1.pro GAA..T...........TI....I......................F...N.........................L...

MF480297_ORF1.pro EAAD.S...A.......TI................I..........Y.................................

MF480298_ORF1.pro GAA..T...........AV...........................F................................V

MG211750_ORF1.pro E.A..T...........DV..................P........H..........S....................V.

MG211751_ORF1.pro E.A..T...........TV...........Y......P........H..........S....................V.

Majority AGAPPPA-GLPHTTPP-VSDVWVLPPPSE------P---PAPEPADPP-------XATP-------------KPPARKPP

---------+---------+---------+---------+---------+---------+---------+---------+

730 740 750 760 770 780 790 800

---------+---------+---------+---------+---------+---------+---------+---------+

DLS11685_ORF1.pro EV.LV.PA.----HT.LAGATSE....L.AAPAGT.LSP.V.R...L.-------A.AQT-------------.V.CGS.

KJ701409_ORF1.pro .A..AA.M....X...-...IR.......ESXVDAAPVP.XS..VQ..G------P.R.-------------.A.V....

KU176129_ORF1.pro VA..AA.P.PR.P...-...I........EFQVDTAPAP......Q.SS------P.G.-------------.A.V....

AB248520_ORF1.pro FA..A.DG...LG..SSA.......S...GSVAASSLAA.VSK..S.LS------P...NPPVHKPLSPPTP........

AB291962_ORF1.pro .P..AA.P..SYP...-...I........ETQVGAVLEP.....VR..S------PVK.-------------P..V....

DLS11677_ORF1.pro .AT.AA.T....P..R-...I........ESQVDAAPAP..L..VQL.G------PVR.-------------.A.V....

DLS11681_ORF1.pro .A..AV.T....S...-...I........ESQADAASAP.V.K.VQ..G------P.G.-------------.A.V....

JQ953664_ORF1.pro .PL.VT.F.P..L...-A..I....S...ESQVTVVPDP.V..HIQ..S------LVK.-------------.TFV..S.

AB073912_ORF1.pro DH..AITS....S...-A..I.A......ESQVDMAPVP...K.VGL.-----SSNEPI--------------T.V....

AB189070_ORF1.pro .P..AA.P.S.PP...-...I........KSQVGA.LAP..L..VG..S------PIKL-------------AS.V....

AB248521_ORF1.pro FA..TFDER..PG..SSL.G..AF.....GSVATPVLAA.VSK..S..S------T...-----K------P--..C...

AB290312_ORF1.pro .PV.TAVL.P..L...-...I........EPQIDVEPAPS...FVQ..S------LVKS-------------.R.V....

AB290313_ORF1.pro FVT.A.DV..S-GP..P..N..IP....GGSAVIPSPET.VSK..NL.S------PVA.-------------........

AB369687_ORF1.pro YV..A.DM...SSA.SSAG...AF.....GSAVVP.PET.VSK..S..I------PT..-------------R..V....

AB369689_ORF1.pro .F.SAA.P.PS.P...-A..I........DTRTSTTLVPSV...VGL.N------PVE.-------------T..V....

AF060668_ORF1.pro .P.MAATP....S...-...I........EFQVDAAPVP...D..GL.GPVVLTPPPP---------------..VH..S

AF082843_ORF1.pro .PVLAA.P....P...-...I.......KESQVDAASVP......GL.SSIVLTLPPPL--------------..V....

AF455784_ORF1.pro .NGE.-----.PGA.S-T.GI........GSEVAL.-TT.VRK.V.L.V------SIP.-------------EA......

AP003430_ORF1.pro .P..AA.P.SSSP...-...I.....L..EPQVGA.PAP.T....RL.C------PTK.-------------NT.V...T

AY115488_ORF1.pro .LPAAAPGP..-P...-...I..V.SL.GESQVDAGLVP......RL.SPTVSTLPSP.-------------P..V....

EU360977_ORF1.pro TA..ALDM.P.SGA.STTG....F.....RSAAVLSPGT.VSK..SL.S------LI..-------------R.......

EU723513_ORF1.pro LV..A.DT...PC..SS.....AF.....GSAIVPLPEA.VSK..NS.I------L...-------------RA.V....

FJ705359_ORF1.pro VA..AATP..R.P...-............EFQVDTAPTP......Q.SS------S.G.-------------.A.V....

FJ998008_ORF1.pro VP..AAIL.P.RS..S-.G.I......F.ESQADTAPPSL.....Q..N------P.KS-------------ET.V.R.S

JQ013794_ORF1.pro .P..VA.L.P..P...-A..I.....L..EHQIDAVPVP......Q..S------PVK.-------------RG.V....

AB740220_ORF1.pro .TV.RSV-D...APT.PGV..QDP...--------L---V..GVVG..-------AN..-------------R.S.H...

AB740221_ORF1.pro ......X-D..C.PA.SEIA.RT....--------.---VDLGVT...-------A..S-------------A...H...

AB740222_ORF1.pro .S.H...-D....PA.SEV..RGPLS.--------H---VV.GAVG..-------AG..-------------GSSVH...

FJ906895_ORF1.pro VSTH...-DR..KPA.SEA..RGPLS.--------L---T..GVVG..-------VS..-------------G.S.H...

FJ906896_ORF1.pro ..V...V-D..SKSA.LGVAAQ.....--------S---VG.GSTG..-------IDA.-------------E.T.Y...

GU937805_ORF1.pro .......-S..C.PA.SEIA.RT....--------.---VDSRVI...-------A..S-------------V.F.H...

JQ013791_ORF1.pro .HT...--SR.YEPA.PEF..QAPS..-------------SVT.S.RS-------I...-------------V.SIPRA.

JQ013792_ORF1.pro VQN..S--DR..HSD.PEHATPAPAL.-------------SIS.SR------------------------------APA.

JQ013793_ORF1.pro VD.....-DP.YGPA.VSAGXRAPL..PL------.---VT.R.TGL.-------AGM.-------------GLLPH...

JQ768461_ORF1.pro .......-S..C.PA.SEIA.RT....--------.---VDSRVI...-------A..S-------------V.F.H...

JX109834_ORF1.pro .......-S..C.PA.SEIA.RT....--------.---VDSRVI...-------A..S-------------V.F.H...

JX121233_ORF1.pro .......-S..C.PA.SEIA.RT....--------.---VDSRVI...-------A..S-------------V.F.H...

JX565469_ORF1.pro ......V-D..SKPA.PEIA.RAPS..--------.---V..G.IG..-------I.A.-------------G.S.H...

KJ013414_ORF1.pro .......-S..C.PA.SEIA.RT....--------.---VDSRVI...-------A..S-------------V.F.H...

KJ013415_ORF1.pro .......-S..C.PA.SEIA.RT....--------.---VDSRVI...-------A..S-------------V.F.H...

KX227751_ORF1.pro ......V-D..Y.PA.SGAA.RGPL..--------.---V..GVVG.S-------AD..-------------G.S.H...

KY436898_ORF1.pro .N....P-T..P.PALPGAGAR.QL..--------S---ET.GC.RLL-------AN..-------------E.STH...

KY496200_ORF1.pro .TV....-DSVQMPT.P.A.ARA...T--------.---VT.RVI...-------A...-------------E...N..S

MF480297_ORF1.pro .N.....-SP...PATSQAR.RA....--------L---EISV..R..-------ADAA-------------R.STH.L.

MF480298_ORF1.pro .NI...S-SA...QA.PEAG.QA....--------L---ETSG.THLL-------ADS.-------------G.SIH...

MG211750_ORF1.pro ..G.S.P-SSS.V.ATP.GGIQ...L.--------S---A..K...R.-------VEV.-------------G.S.H...

MG211751_ORF1.pro ..G.S.P-SSS.V.ATP.GGIQ...L.--------S---A..K...R.-------VEV.-------------G.S.H...

Majority TPPPARNRRLLYTYPDGAKVYAGSLFESDCDWLVNASNPGHRPGGGLCHAFYQRFPESFDPTKFIMRDGLAAYTLTPRPI

---------+---------+---------+---------+---------+---------+---------+---------+

810 820 830 840 850 860 870 880

---------+---------+---------+---------+---------+---------+---------+---------+

DLS11685_ORF1.pro A...T.T.....A....SR........................................RS.E....E............

KJ701409_ORF1.pro ....PXT.X.X...........................................Y....YS.E.................

KU176129_ORF1.pro V..SP.T...............................................Y....H..E.................

AB248520_ORF1.pro P.....T....................................................H..D....E............

AB291962_ORF1.pro ALS.S.A.......................Y..........................A.Y.AD....E............

DLS11677_ORF1.pro ....P.T...............................................Y.A..H..E....E............

DLS11681_ORF1.pro ....P.T...............................................Y....Y..E....E............

JQ953664_ORF1.pro ....S.T...............................................Y....Y..E.V...............

AB073912_ORF1.pro A..AS.T..................................................A.Y..E....E............

AB189070_ORF1.pro .L..S.T..................................................A.Y..E....E............

AB248521_ORF1.pro P...T.T...............................................Y....H..D....E............

AB290312_ORF1.pro ....S.T.........................................L..........H..E..T..............

AB290313_ORF1.pro A..SV.T....................................................H..D.V..E.......S....

AB369687_ORF1.pro ..S.T.T.....................X.........................Y....H..D.V..E.......S....

AB369689_ORF1.pro A...P.T..................................................A.Y..E....E............

AF060668_ORF1.pro I...S....................................................A.Y..E....E............

AF082843_ORF1.pro ....S.T.......................N..........................A.Y..E....E............

AF455784_ORF1.pro A.....T....................................................Y.AD.................

AP003430_ORF1.pro A...S.T..................................................A.Y..E....E............

AY115488_ORF1.pro A..LP.T..................................................A.YS.E....E............

EU360977_ORF1.pro ...........H...............................................H..D.V..E.......C....

EU723513_ORF1.pro ......T....................................................Y.AD.V..E.......S....

FJ705359_ORF1.pro ...SP.T...............................................Y....H..E.................

FJ998008_ORF1.pro .QLLH.T...............................................Y....H..E.................

JQ013794_ORF1.pro ....S.T.....................S.........................Y....H..E.................

AB740220_ORF1.pro G.SL...............I.S..................................Q...H.....Q.............

AB740221_ORF1.pro GRSS.................S..................................Q...H..............S....

AB740222_ORF1.pro G.CS...............I.S..................................Q...H...................

FJ906895_ORF1.pro G.SS...............I.S..................................Q...H...................

FJ906896_ORF1.pro AQ..V.......I...................P.......................Q...H..............S....

GU937805_ORF1.pro GRSS.................S................S.................Q...H..............L....

JQ013791_ORF1.pro VQAVV......H.........S..................................Q.......V...............

JQ013792_ORF1.pro PQVTV......H.........S..................................Q....A..V...............

JQ013793_ORF1.pro .Q..........V......I.S..................................Q...H...T...............

JQ768461_ORF1.pro ERSS.................S..................................Q...H..............L....

JX109834_ORF1.pro ERSS.................S..................................Q...H..............L....

JX121233_ORF1.pro ERSS.................S..................................Q...H..............L....

JX565469_ORF1.pro AQAS.................S..................................Q...H...................

KJ013414_ORF1.pro ERSS.................S..................................Q...H..............L....

KJ013415_ORF1.pro ERSS.................S..................................Q...H..............L....

KX227751_ORF1.pro G.SL.................S..................................Q...H...V...............

KY436898_ORF1.pro ER.SV.S............I.S.............................H....Q...H..............S....

KY496200_ORF1.pro GR...................S..................................L...H..............S....

MF480297_ORF1.pro GQS................I.S....D.............................Q...H..............S....

MF480298_ORF1.pro GR.L...............I.S..................................Q...HS.............S....

MG211750_ORF1.pro GH.................I.S.......................................A..V.........FS....

MG211751_ORF1.pro GH.................I.S.......................................A..V..........S....

Majority IHAVAPDYRVEHNPKRLEAAYRETCSRLGTAAYPLLGSGIYQVPVGLSFDAWERNHRPGDELYLTEPAAAWFEANKPAQ-

---------+---------+---------+---------+---------+---------+---------+---------+

890 900 910 920 930 940 950 960

---------+---------+---------+---------+---------+---------+---------+---------+

DLS11685_ORF1.pro .........L.................R.........A.......S......................I........S.-

KJ701409_ORF1.pro ...........Q...............R.................S...................DL..T.........-

KU176129_ORF1.pro ...........................R.................S...................DL..T.......T.-

AB248520_ORF1.pro .........I.Q.................................................................T.-

AB291962_ORF1.pro ...........Q...............R...................................................-

DLS11677_ORF1.pro ...........Q...............R.................S...................D...T.........-

DLS11681_ORF1.pro ...........Q...............R.................S...................DL............-

JQ953664_ORF1.pro ...........Q...............R.....................................D..V........T.-

AB073912_ORF1.pro ...........Q...............R.................S.................................-

AB189070_ORF1.pro ...........Q...............R...................................................-

AB248521_ORF1.pro ...........Q.................................................................T.-

AB290312_ORF1.pro ...........Q...............R.....................................D...........T.-

AB290313_ORF1.pro ...........Q........................E........................................T.-

AB369687_ORF1.pro ...........Q.................................................................T.-

AB369689_ORF1.pro .........A.Q...............R.............R...S......................V........M.-

AF060668_ORF1.pro ...........Q...............R.................S.......................N.........-

AF082843_ORF1.pro ...........Q...............R.................S.................................-

AF455784_ORF1.pro ...........Q...............R...................................................-

AP003430_ORF1.pro ...........Q...............R................A................................T.-

AY115488_ORF1.pro ...........Q...............R.................S...............................M.-

EU360977_ORF1.pro ...........Q.................................S...............................T.-

EU723513_ORF1.pro ...........Q...................................................................-

FJ705359_ORF1.pro ...........................R.................S...................DL..T.......T.-

FJ998008_ORF1.pro ...........Q...............R.....................................D...T.......T.-

JQ013794_ORF1.pro ...........Q...............R.................S...................D...........T.-

AB740220_ORF1.pro ..........A...................................P...................L.T.........RP

AB740221_ORF1.pro ..........A...................................P.....................I.........RX

AB740222_ORF1.pro ..........A...................................P...................L.T......R..RS

FJ906895_ORF1.pro ..........AN................S.................P...................L.T......R.TRS

FJ906896_ORF1.pro ..........A................R..................P..................D............RP

GU937805_ORF1.pro ..........A...................................P.....................T.........RP

JQ013791_ORF1.pro ..........A...................................P.....................V......R..RS

JQ013792_ORF1.pro .........AA...................................P...................L.T......R..RS

JQ013793_ORF1.pro ..........A...................................P.....................T......R..RL

JQ768461_ORF1.pro ..........A...................................P.....................T.........RP

JX109834_ORF1.pro ..........A...................................P.....................T.........RP

JX121233_ORF1.pro ..........A..................................DP.....................T.........RP

JX565469_ORF1.pro ..........A...................................P.....................T......R..RP

KJ013414_ORF1.pro ..........A...................................P.....................T.........RP

KJ013415_ORF1.pro ..........A...................................P.....................T.........RP

KX227751_ORF1.pro ..........A...................................P...................L.T......R..RP

KY436898_ORF1.pro ..........A................................................................R..RS

KY496200_ORF1.pro ..........A...................................P...............................RS

MF480297_ORF1.pro ..........A...................................P...................L.T......R..RS

MF480298_ORF1.pro .........AA...................................P.....................T......R..RP

MG211750_ORF1.pro ...........................F..................P.....................VT.....R.VRS

MG211751_ORF1.pro ..............................................P.....................VT.....R.VRS

Majority ------------------------------PALTITEDTARTANLALEIDSATEVGRACAGCTVSPGVVHYQFTAGVPGS

---------+---------+---------+---------+---------+---------+---------+---------+

970 980 990 1000 1010 1020 1030 1040

---------+---------+---------+---------+---------+---------+---------+---------+

DLS11685_ORF1.pro ------------------------------...S....V..............D.......LS.................

KJ701409_ORF1.pro ------------------------------....................A................I............

KU176129_ORF1.pro ------------------------------........A...........A...........A.................

AB248520_ORF1.pro ------------------------------.............................T.......I............

AB291962_ORF1.pro ------------------------------...M................A...........I.N...............

DLS11677_ORF1.pro ------------------------------....................A.............................

DLS11681_ORF1.pro ------------------------------....................A...........A....I............

JQ953664_ORF1.pro ------------------------------....................A.............................

AB073912_ORF1.pro ------------------------------....................A............IR...............

AB189070_ORF1.pro ------------------------------....................A............I...I............

AB248521_ORF1.pro ------------------------------................................V.N...............

AB290312_ORF1.pro ------------------------------....................AT...............I............

AB290313_ORF1.pro ------------------------------................................S.N..I............

AB369687_ORF1.pro ------------------------------.................................IN..I............

AB369689_ORF1.pro ------------------------------....................A.............................

AF060668_ORF1.pro ------------------------------.V..................A............I...I............

AF082843_ORF1.pro ------------------------------....................A..D.........I...I............

AF455784_ORF1.pro ------------------------------........I............................I............

AP003430_ORF1.pro ------------------------------....................A..D.........I...I............

AY115488_ORF1.pro ------------------------------....................A............I...I............

EU360977_ORF1.pro ------------------------------................................AIN...............

EU723513_ORF1.pro ------------------------------..................................N...............

FJ705359_ORF1.pro ------------------------------........A...........A...........A.................

FJ998008_ORF1.pro ------------------------------....................A................I............

JQ013794_ORF1.pro ------------------------------....................A.............................

AB740220_ORF1.pro PPPTTHDTASADHLAAGAGLSLGAGAAPAL...........A.........K..L..............R..........

AB740221_ORF1.pro PTPIVLDMPLPDCLPAGAGSPPEADTGPAL........A............R..L.......A......R..........

AB740222_ORF1.pro LTPTAHDTASAEHPVAGAGLSPGAETGPVL........I............R..L.......SI.....R..........

FJ906895_ORF1.pro LTPTTHDTASADYPATGAGLSPGADTGPAL.....................R..L........I.....R..........

FJ906896_ORF1.pro PTPIVHDMAPADHPVAGAGPPPQANTSPAL........A............R..L....T..V.N....R.R........

GU937805_ORF1.pro PIPIVLDTPPPDHPPTGAGSPPEADTGPAL........A............R..L.......A......R..........

JQ013791_ORF1.pro PVPVADDVTRTAGPVMGPSPSVDAAGGSAL...V....A........K...G.................Y..........

JQ013792_ORF1.pro PAPTIDVAAPTAGPVPGVSSPAEAVGEPAL...V....A........K.......................K........

JQ013793_ORF1.pro STLTTQDIAPADSPAAGTDPSPEPARGPAL.....................R..L.........N...............

JQ768461_ORF1.pro PIPIVLDTPPPDHPPTGAGSPPEADTGPAL........A............R..L.......A......R..........

JX109834_ORF1.pro PIPIVLDTPPPDHPPTGAGSPPEADTGPAL........A............R..L.......A......R..........

JX121233_ORF1.pro PIPIVLDTPPPDHPPTGAGSPPEADTGPAL........A............R..L.......A......R..........

JX565469_ORF1.pro PTPTTHDTAPADHPATGAGPRPEADTGPAL........A............R..L..............R..........

KJ013414_ORF1.pro PIPIVLDTPPPDHPPTGAGSPPEADTGPAL........A............R..L.......A......R..........

KJ013415_ORF1.pro PIPIVLDTPPPDHPPTGAGSPPEADTGPAL........A............K..L.......A......R..........

KX227751_ORF1.pro LTPTTHDTAPADYPVAGAGPPSGAGAGPVL........I............R..L..............R..........

KY436898_ORF1.pro FTPAAPGTASAGGPSVGTGPSPNTAGVPAL........V........A...K..L.........N...IY..........

KY496200_ORF1.pro STPID-DTASADRPAAGADSPPGVGTGPVL........A..V.........K..L.......A.................

MF480297_ORF1.pro LIPVTPGTALADDPAVVVGPMPEADDGPEL.....................K..L.......A.................

MF480298_ORF1.pro LIPAVSDMAPADDPAVGAGPQPEAADGPEL........A............R..L....V..A......R..........

MG211750_ORF1.pro SMPMTNDPPLAADPPSGADRPPGADGHPAL......D.I............R.......T...L................

MG211751_ORF1.pro SMPMTNDPPLAADPPSGADRPPGADGHPAL......D.I............R.......T...L................

Majority GKSRSIQQGDVDVVVVPTRELRNSWRRRGFAAFTPHTAARVTSGRRVVIDEAPSLPPHLLLLHMQRASSVHLLGDPNQIP

---------+---------+---------+---------+---------+---------+---------+---------+

1050 1060 1070 1080 1090 1100 1110 1120

---------+---------+---------+---------+---------+---------+---------+---------+

DLS11685_ORF1.pro .........E......................Y.........G...........V.........................

KJ701409_ORF1.pro ..........................................T.....................................

KU176129_ORF1.pro ..........................................T.....................................

AB248520_ORF1.pro ..........................................A.....................................

AB291962_ORF1.pro ..........................................T.....................................

DLS11677_ORF1.pro ..........................................T.....................................

DLS11681_ORF1.pro ..........................................T.....................................

JQ953664_ORF1.pro .......................C........................................................

AB073912_ORF1.pro ..........................................T.....................................

AB189070_ORF1.pro ..........................................I.....................................

AB248521_ORF1.pro ..........................................A.....................................

AB290312_ORF1.pro ..........................................L.....................................

AB290313_ORF1.pro ..........................................A.............L.......................

AB369687_ORF1.pro ..........................................A.....................................

AB369689_ORF1.pro ..........................................I.....................................

AF060668_ORF1.pro ..........................................I.....................................

AF082843_ORF1.pro ..........................................I.....................................

AF455784_ORF1.pro ..........................................G.....................................

AP003430_ORF1.pro ..........................................I.....................................

AY115488_ORF1.pro ..........................................T.....................................

EU360977_ORF1.pro ..........................................A.........................T...........

EU723513_ORF1.pro ..........................................A........................A............

FJ705359_ORF1.pro ..........................................T.....................................

FJ998008_ORF1.pro ..........................................A.....................................

JQ013794_ORF1.pro ..........................................L.....................................

AB740220_ORF1.pro ......................G.............S...........................................

AB740221_ORF1.pro ....................................S...........................................

AB740222_ORF1.pro ......................GN............S...........................................

FJ906895_ORF1.pro ......................GN............S...........................................

FJ906896_ORF1.pro ............................R.......S...........................................

GU937805_ORF1.pro .......R............................S..................................X........

JQ013791_ORF1.pro ...................................................................L........H...

JQ013792_ORF1.pro ..........................................C.....................................

JQ013793_ORF1.pro ....................................S...........................................

JQ768461_ORF1.pro ....................................S...........................................

JX109834_ORF1.pro ....................................S...........................................

JX121233_ORF1.pro ....................................S...........................................

JX565469_ORF1.pro ....................................S...........................................

KJ013414_ORF1.pro ....................................S...........................................

KJ013415_ORF1.pro ....................................S...........................................

KX227751_ORF1.pro ......................G.............S...........................................

KY436898_ORF1.pro ....................................S....SG.....................................

KY496200_ORF1.pro ....................................S...........................................

MF480297_ORF1.pro ....................................S.....A.....................................

MF480298_ORF1.pro ....................................S.....G........................A............

MG211750_ORF1.pro ....................................S.....G.....................................

MG211751_ORF1.pro ....................................S.....G.....................................

Majority AIDFEHAGLVPAIRPELAPTSWWHVTHRCPADVCELIRGAYPKIQTTSRVLRSLFWNEPAVGQKLVFTQAAKAANPGAIT

---------+---------+---------+---------+---------+---------+---------+---------+

1130 1140 1150 1160 1170 1180 1190 1200

---------+---------+---------+---------+---------+---------+---------+---------+

DLS11685_ORF1.pro .................V..................V.......................S...................

KJ701409_ORF1.pro ......................................................Y.....I...................

KU176129_ORF1.pro ............................................................I...................

AB248520_ORF1.pro ......S...........................................M.............................

AB291962_ORF1.pro ............................................................I...................

DLS11677_ORF1.pro ............................................................I...................

DLS11681_ORF1.pro ............................................................I...................

JQ953664_ORF1.pro ............................................................T...................

AB073912_ORF1.pro ............................................................I...................

AB189070_ORF1.pro ....................T.......................................I...................

AB248521_ORF1.pro ....................A.............................M.............................

AB290312_ORF1.pro ............................................................I...................

AB290313_ORF1.pro ..................L.......................................S...R.................

AB369687_ORF1.pro ................................................................................

AB369689_ORF1.pro ............................................................I...................

AF060668_ORF1.pro .......................X....................................I.....X.............

AF082843_ORF1.pro ............................................................I...................

AF455784_ORF1.pro ............................................................I...................

AP003430_ORF1.pro ............................................................I...................

AY115488_ORF1.pro ..................................................R.........S...................

EU360977_ORF1.pro ................................................................................

EU723513_ORF1.pro ................................................................................

FJ705359_ORF1.pro ............................................................I...................

FJ998008_ORF1.pro ............................................................I...................

JQ013794_ORF1.pro ............................................................T...................

AB740220_ORF1.pro ......T.................................................GD.P...R................

AB740221_ORF1.pro ....................................V...................GD.P....................

AB740222_ORF1.pro ........................................................G..P............V.......

FJ906895_ORF1.pro ........................................................G..P....................

FJ906896_ORF1.pro ........................................................GD.P....................

GU937805_ORF1.pro .................................X......................GD.P....................

JQ013791_ORF1.pro .....NT.................................................GD.PI...................

JQ013792_ORF1.pro ............L...............................H...........GD.P....................

JQ013793_ORF1.pro ........................................................GD.P...R.............S..

JQ768461_ORF1.pro ........................................................GD.P....................

JX109834_ORF1.pro ........................................................GD.P....................

JX121233_ORF1.pro ........................................................GD.P....................

JX565469_ORF1.pro ....................................V...................GD.P....................

KJ013414_ORF1.pro ........................................................GD.P....................

KJ013415_ORF1.pro ........................................................GD.P....................

KX227751_ORF1.pro ........................................................GD.P....................

KY436898_ORF1.pro ........................................................GD.PT...................

KY496200_ORF1.pro ........................................................GD.P.............VY.....

MF480297_ORF1.pro ........................................................GD.PT...................

MF480298_ORF1.pro ......T.............................V...................GH.PI...................

MG211750_ORF1.pro ....................X...................................G..PI...................

MG211751_ORF1.pro ........................................................G..PI..................X

Majority VHEAQGATFTETTIIATADARGLIQSSRAHAIVALTRHTEKCVILDAPGLLREVGISDVIVNNFFLAGGEVGHHRPSVIP

---------+---------+---------+---------+---------+---------+---------+---------+

1210 1220 1230 1240 1250 1260 1270 1280

---------+---------+---------+---------+---------+---------+---------+---------+

DLS11685_ORF1.pro ..........V...............................................IM.K..................

KJ701409_ORF1.pro ..........................................................I.....................

KU176129_ORF1.pro ................................................................................

AB248520_ORF1.pro ........................................R.................I..................T..

AB291962_ORF1.pro ................................................................................

DLS11677_ORF1.pro ..........V...............................................I.....................

DLS11681_ORF1.pro ..........................................................I.....................

JQ953664_ORF1.pro ..........................................................I.....................

AB073912_ORF1.pro ..........................................I..............................N......

AB189070_ORF1.pro ........................................R.......................................

AB248521_ORF1.pro ..........................................................I..................T..

AB290312_ORF1.pro .............................Y............................I.....................

AB290313_ORF1.pro ..........................................................I..................T..

AB369687_ORF1.pro .......................................G..................I..................T..

AB369689_ORF1.pro ................................................................................

AF060668_ORF1.pro ........................................................................X.......

AF082843_ORF1.pro ................................................................................

AF455784_ORF1.pro ..........................................................I..................T..

AP003430_ORF1.pro ................................................................................

AY115488_ORF1.pro ..........................................I..........I..........................

EU360977_ORF1.pro ..........................................................I..................T..

EU723513_ORF1.pro ..........................................................I..................I..

FJ705359_ORF1.pro ................................................................................

FJ998008_ORF1.pro .............V............................................I.....................

JQ013794_ORF1.pro ..........................................................I.....................

AB740220_ORF1.pro ......................................................................I.........

AB740221_ORF1.pro ......................................................................I.........

AB740222_ORF1.pro ......................................................................I.........

FJ906895_ORF1.pro .....................D...........................................I....I.......V.

FJ906896_ORF1.pro ................................A.....................................I.........

GU937805_ORF1.pro ......................................................................I.........

JQ013791_ORF1.pro ..........................................................A.....................

JQ013792_ORF1.pro .................S........................................A...........I.........

JQ013793_ORF1.pro ................................................................................

JQ768461_ORF1.pro ......................................................................I.........

JX109834_ORF1.pro ......................................................................I.........

JX121233_ORF1.pro ......................................................................I.........

JX565469_ORF1.pro ......................................................................I.........

KJ013414_ORF1.pro ......................................................................I.........

KJ013415_ORF1.pro ......................................................................I.........

KX227751_ORF1.pro ......................................................................I.........

KY436898_ORF1.pro ............................................I.........................I.........

KY496200_ORF1.pro .......................................................V..............I.........

MF480297_ORF1.pro ............................................I.........................I.........

MF480298_ORF1.pro .............V..............................I.............A..S........I.........

MG211750_ORF1.pro ........Y.............................................................I.........

MG211751_ORF1.pro ........Y.............................................................I.........

Majority RGNPDQNLDTLQAFPPSCQISAYHQLAEELGHRPAPVAAVLPPCPELEQGLLYMPQELTVSDSVLVFELTDIVHCRMAAP

---------+---------+---------+---------+---------+---------+---------+---------+

1290 1300 1310 1320 1330 1340 1350 1360

---------+---------+---------+---------+---------+---------+---------+---------+

DLS11685_ORF1.pro ................................................................................

KJ701409_ORF1.pro ..S.....A.......................................................................

KU176129_ORF1.pro ....X...X....................X..X...............................................

AB248520_ORF1.pro .........I......................................................................

AB291962_ORF1.pro .....K..........................................................................

DLS11677_ORF1.pro ..S.....A.......................................................................

DLS11681_ORF1.pro ..S.....A...............................TA.L....................................

JQ953664_ORF1.pro ........A.......................................................................

AB073912_ORF1.pro .......VE.......................................................................

AB189070_ORF1.pro ........E.......................................................................

AB248521_ORF1.pro ................................................................................

AB290312_ORF1.pro ..S.............................................................................

AB290313_ORF1.pro ..Y........R........................M............................I....N.........

AB369687_ORF1.pro ...........R....................................................................

AB369689_ORF1.pro ................................................................................

AF060668_ORF1.pro ........G.......................................................................

AF082843_ORF1.pro ........G.......................................................................

AF455784_ORF1.pro .....R....................................................A.....................

AP003430_ORF1.pro .....R..........................................................................

AY115488_ORF1.pro ........G..............Y..............................................NM.....P..

EU360977_ORF1.pro ...........R....................................................................

EU723513_ORF1.pro .................................................................I..............

FJ705359_ORF1.pro ........A.......................................................................

FJ998008_ORF1.pro ....G...E.......................................................................

JQ013794_ORF1.pro ..S.G...E.......................................................................

AB740220_ORF1.pro .....R.F..............F.........................................................

AB740221_ORF1.pro .....R...I......................................................................

AB740222_ORF1.pro .....R................F.........................................................

FJ906895_ORF1.pro H....R................F......................................N..................

FJ906896_ORF1.pro .....R...I.....................................................I................

GU937805_ORF1.pro .....R...I......................................................................

JQ013791_ORF1.pro ........E..............................L........................................

JQ013792_ORF1.pro ........EI.............................L..................A.....................

JQ013793_ORF1.pro .....R..........................................................................

JQ768461_ORF1.pro .....R...I.........................................................G............

JX109834_ORF1.pro .....R...I.........................................................G............

JX121233_ORF1.pro .....R...I......................................................................

JX565469_ORF1.pro .....R................F.........................................................

KJ013414_ORF1.pro .....R...I......................................................................

KJ013415_ORF1.pro .....R...I......................................................................

KX227751_ORF1.pro .....R...I............F.........................................................

KY436898_ORF1.pro ........E.......................................................................

KY496200_ORF1.pro .....R..........................................................................

MF480297_ORF1.pro .....P..Q..E....................................................................

MF480298_ORF1.pro .....R..E.......................................................................

MG211750_ORF1.pro .....P..A.......................................................................

MG211751_ORF1.pro .....P..A...........................L...........................................

Majority SQRKAVLSTLVGRYGRRTKLYEAAHSDVRESLARFIPTIGPVQATTCELYELVEAMVEKGQDGSAVLELDLCNRDVSRIT

---------+---------+---------+---------+---------+---------+---------+---------+

1370 1380 1390 1400 1410 1420 1430 1440

---------+---------+---------+---------+---------+---------+---------+---------+

DLS11685_ORF1.pro ................................................................................

KJ701409_ORF1.pro ................................................................................

KU176129_ORF1.pro ................................................................................

AB248520_ORF1.pro ................................G.......................................S.......

AB291962_ORF1.pro ................K...............................................................

DLS11677_ORF1.pro ................................T........................D......................

DLS11681_ORF1.pro .........................................................D......................

JQ953664_ORF1.pro ................................................................................

AB073912_ORF1.pro ..........................................R.....................................

AB189070_ORF1.pro ................K...............................................................

AB248521_ORF1.pro ................................GK...S..................................S.......

AB290312_ORF1.pro .............C..K...............................................P...............

AB290313_ORF1.pro ....................N...........G.......................................S.......

AB369687_ORF1.pro ................................G.......................................S.......

AB369689_ORF1.pro ................K...............................................................

AF060668_ORF1.pro ................................................................................

AF082843_ORF1.pro ................................................................................

AF455784_ORF1.pro .................................K...............C......................S.......

AP003430_ORF1.pro ................K...............................................................

AY115488_ORF1.pro ...........E....................................................................

EU360977_ORF1.pro ................................G.......................................S.......

EU723513_ORF1.pro ................................G.......................................S.......

FJ705359_ORF1.pro ................................................................................

FJ998008_ORF1.pro ................................................................................

JQ013794_ORF1.pro ................................................................................

AB740220_ORF1.pro ................................T.......................................S.......

AB740221_ORF1.pro ..................R.............S....A..................................S.......

AB740222_ORF1.pro ................................T....I..........................................

FJ906895_ORF1.pro ................................T....I..........................................

FJ906896_ORF1.pro ..................R.............M...................................P...S.......

GU937805_ORF1.pro ..................R......T......S....A..................................S.....V.

JQ013791_ORF1.pro ................................T...............................................

JQ013792_ORF1.pro .....................D..........T...............................................

JQ013793_ORF1.pro ..................R.....................................................S.......

JQ768461_ORF1.pro ..................R......A......S....A..............................P...S.......

JX109834_ORF1.pro ..................R......A......S....A..............................P...S.......

JX121233_ORF1.pro ..................R......A......S....A..................................S.......

JX565469_ORF1.pro ................................T.......................................S.......

KJ013414_ORF1.pro ..................R......A......S....A...............Q..................S.......

KJ013415_ORF1.pro ..................R......A......S....A...............Q..............P...S.......

KX227751_ORF1.pro ................................T.......................................S.......

KY436898_ORF1.pro ..................R..D..........T....A..........................................

KY496200_ORF1.pro .....I..........................T.......................................S.......

MF480297_ORF1.pro .................SR.............T.......................................S.......

MF480298_ORF1.pro .................SR.............T...AI....................................ILCLV.

MG211750_ORF1.pro ..................R....T........T.......................................S.......

MG211751_ORF1.pro ..................R..D.T........T.......................................S.......

Majority FFQKDCNKFTTGETIAHGKVGQGISAWSKTFCALFGPWFRAIEKEILALLPPNIFYGDAYEXSVFSAAVSGAGSSMVFEN

---------+---------+---------+---------+---------+---------+---------+---------+

1450 1460 1470 1480 1490 1500 1510 1520

---------+---------+---------+---------+---------+---------+---------+---------+

DLS11685_ORF1.pro ................................................Q....V.......D......IA..S.C.....

KJ701409_ORF1.pro ...................................................X.........E...A........C.....

KU176129_ORF1.pro .............................................................E...A..............

AB248520_ORF1.pro ...............................................S...........F.E......I...........

AB291962_ORF1.pro ...............................................T.............E...A........C.....

DLS11677_ORF1.pro .............................................................E...A........C.....

DLS11681_ORF1.pro .............................................................E...A........C.....

JQ953664_ORF1.pro ...........................................................F.E...A........C.....

AB073912_ORF1.pro .............................................................G...A........C.....

AB189070_ORF1.pro .............................................................E...A........C.....

AB248521_ORF1.pro ...........................................................F.E..................

AB290312_ORF1.pro .............................................................E...A........C.....

AB290313_ORF1.pro .............................................................E......I.....C.....

AB369687_ORF1.pro .....................................................V.......E......I...........

AB369689_ORF1.pro .............................................................E...A........C.....

AF060668_ORF1.pro ....X........................................................E...A........C.....

AF082843_ORF1.pro .............................................................E...A........C.....

AF455784_ORF1.pro .............................................................E...........T......

AP003430_ORF1.pro .............................................................E...A........C.....

AY115488_ORF1.pro .............................................................E...A........C.....

EU360977_ORF1.pro .............................................................E......I...........

EU723513_ORF1.pro .............................................................E......I....T......

FJ705359_ORF1.pro .............................................................E...A..............

FJ998008_ORF1.pro ...............................................T.............E...A........C.....

JQ013794_ORF1.pro .............................................................E...A........C.....

AB740220_ORF1.pro ............................................Q..............F.D.......T..........

AB740221_ORF1.pro ............................................Q..T.............D.......T..........

AB740222_ORF1.pro ............................................Q................D.......T..........

FJ906895_ORF1.pro ..............................L.............Q...............GD....V..T..........

FJ906896_ORF1.pro ...........................G................Q................D.......T..........

GU937805_ORF1.pro ............................................P..T.............D.......T..........

JQ013791_ORF1.pro ...............................................T.....V.......D.......T..........

JQ013792_ORF1.pro ...............................................T.............D.......T..........

JQ013793_ORF1.pro ............................................Q................D.......T..........

JQ768461_ORF1.pro ............................................Q..T...........H.D.......T..........

JX109834_ORF1.pro ............................................Q..T...........H.D.......T..........

JX121233_ORF1.pro ............................................Q..T.............D.......T..........

JX565469_ORF1.pro ....................A.......................KVIL.............D.......T..........

KJ013414_ORF1.pro ............................................Q..T.............D.......T..........

KJ013415_ORF1.pro ............................................Q..T.............D.......T..........

KX227751_ORF1.pro ............................................Q................D.......T..........

KY436898_ORF1.pro ............................................Q........V.......D.......T.S........

KY496200_ORF1.pro ............................................Q................D.....T.T..........

MF480297_ORF1.pro ............................................Q..T.............DT......T..........

MF480298_ORF1.pro VT.......P........RSCV.T.SRY.......RMGH.....QV.............F.D.......T.G........

MG211750_ORF1.pro ............................................Q................D.......T..V.......

MG211751_ORF1.pro ............................................Q................DF......TX.V.......

Majority DFSEFDSTQNNFSLGLECVIMEECGMPQWLIRLYHLVRSAWILQAPKESLKGFWKKHSGEPGTLLWNTVWNMAIIAHCYE

---------+---------+---------+---------+---------+---------+---------+---------+

1530 1540 1550 1560 1570 1580 1590 1600

---------+---------+---------+---------+---------+---------+---------+---------+

DLS11685_ORF1.pro ..................A............G........................L................V......

KJ701409_ORF1.pro ...X...............V.............X......................................X.......

KU176129_ORF1.pro ...................V............................................................

AB248520_ORF1.pro .........................................T......................................

AB291962_ORF1.pro ...................V................................................I...........

DLS11677_ORF1.pro ...................V............................................................

DLS11681_ORF1.pro ...................V...WSL..T...................................................

JQ953664_ORF1.pro ..............................V..........V......................................

AB073912_ORF1.pro ...................V............................................................

AB189070_ORF1.pro ...................V............................................................

AB248521_ORF1.pro .........................................T......................................

AB290312_ORF1.pro ...................V............................................................

AB290313_ORF1.pro ....................................A....T......................................

AB369687_ORF1.pro .........................................T......................................

AB369689_ORF1.pro .........................................V......................................

AF060668_ORF1.pro ...................V............................................................

AF082843_ORF1.pro ...................V............................................................

AF455784_ORF1.pro ..............................V..........T......................................

AP003430_ORF1.pro ...................V................................................I...........

AY115488_ORF1.pro ...................V................................................I...........

EU360977_ORF1.pro .........................................T..........................I...........

EU723513_ORF1.pro .........................................T......................................

FJ705359_ORF1.pro ...................V............................................................

FJ998008_ORF1.pro ...................V............................................................

JQ013794_ORF1.pro ..................XV................................................I...........

AB740220_ORF1.pro ...........................P..V..........V..........L....................V......

AB740221_ORF1.pro ..............................V..........................................V......

AB740222_ORF1.pro ..............................V..........................................V......

FJ906895_ORF1.pro ..............................V..........................................V......

FJ906896_ORF1.pro .........................................................................V......

GU937805_ORF1.pro ..............................V..........................................V......

JQ013791_ORF1.pro ..............................V...........W.........................I......S....

JQ013792_ORF1.pro ..............................V.....................................I....V.S....

JQ013793_ORF1.pro ..............................V..........................................V......

JQ768461_ORF1.pro ..............................V..........................................V......

JX109834_ORF1.pro ..............................V..........................................V......

JX121233_ORF1.pro ..............................V..........................................V......

JX565469_ORF1.pro ..............................V........S.................................V......

KJ013414_ORF1.pro ..............................V...Q......................................V......

KJ013415_ORF1.pro ..............................V...Q......................................V......

KX227751_ORF1.pro ..............................V.....................................I....V......

KY436898_ORF1.pro ..............................V..........................................V......

KY496200_ORF1.pro ..............................V..........................................V......

MF480297_ORF1.pro ...................L...........K.SQ......................................V......

MF480298_ORF1.pro .......................S..R.R.V.....................................I....V......

MG211750_ORF1.pro ...............................K................................................

MG211751_ORF1.pro ..............S................K................................................

4794 nt (aa 1652)

Majority FRDFRVAAFKGDDSVVLCSDYRQSRNAAALIAGCGLKLKVDYRPIGLYAGVVVAPGLGTLPDVVRFAGRLSEKNWGPGPE

---------+---------+---------+---------+---------+---------+---------+---------+

1610 1620 1630 1640 1650 1660 1670 1680

---------+---------+---------+---------+---------+---------+---------+---------+

DLS11685_ORF1.pro ...L...........I.........H..........R.......V.............A.....................

KJ701409_ORF1.pro ................................................................................

KU176129_ORF1.pro ...L............................................................................

AB248520_ORF1.pro ...L..............T.......................................A.....................

AB291962_ORF1.pro ............................T...................................................

DLS11677_ORF1.pro ................................................................................

DLS11681_ORF1.pro ....K...........................................................................

JQ953664_ORF1.pro ................................................................................

AB073912_ORF1.pro ...L............................................................................

AB189070_ORF1.pro ...L..............T.........T...................................................

AB248521_ORF1.pro ...L.M....................................................A...I.................

AB290312_ORF1.pro .........................................................................D......

AB290313_ORF1.pro ...M......................................................A.....................

AB369687_ORF1.pro ...L........................T...............V.............A.....................

AB369689_ORF1.pro ..................N..........................................................S..

AF060668_ORF1.pro ................................................................................

AF082843_ORF1.pro ................................................................................

AF455784_ORF1.pro ....K...............................................I.....A.....................

AP003430_ORF1.pro ................................................................................

AY115488_ORF1.pro ..E.............................................................................

EU360977_ORF1.pro ...L........................T.............................A.....................

EU723513_ORF1.pro ...L......................................................A...................L.

FJ705359_ORF1.pro ................................................................................

FJ998008_ORF1.pro ................................................................................

JQ013794_ORF1.pro ................................................................................

AB740220_ORF1.pro ....K....................F..................V.........................T.........

AB740221_ORF1.pro ....K....................F..................V...................................

AB740222_ORF1.pro .........................F..T...............V.................I.................

FJ906895_ORF1.pro .........................F.T................V.................I...........R.....

FJ906896_ORF1.pro ....K....................F..................V................................S..

GU937805_ORF1.pro ....K....................F..................V...................................

JQ013791_ORF1.pro ....K.............G......F..................V...................................

JQ013792_ORF1.pro ....KA............G......F..T...............V.............V.....................

JQ013793_ORF1.pro ...L.A...................F........................I.............................

JQ768461_ORF1.pro ....K....................F..................V...................................

JX109834_ORF1.pro ....K....................F..................V...................................

JX121233_ORF1.pro ....K....................F..................V...................................

JX565469_ORF1.pro .........................F................................A.....................

KJ013414_ORF1.pro ....K....................F..................V...................................

KJ013415_ORF1.pro ....K....................F..................V...................................

KX227751_ORF1.pro .........................F..................V...................................

KY436898_ORF1.pro ....KA...................F......................................................

KY496200_ORF1.pro ....K....................F..................V...................................

MF480297_ORF1.pro ....K....................F...............F..A.......I...........................

MF480298_ORF1.pro ....KA.....N.............F....V..........F......................................

MG211750_ORF1.pro ....K....................L......................................................

MG211751_ORF1.pro ...XK....................L......................................................

Majority RAEQLRLAVCDFLRKLTNVAQVCVDVVSRVYGVSPGLVHNLIGMLQTIADGKAHFTETVKPVLDLTNSIIQRLE-

---------+---------+---------+---------+---------+---------+---------+-----

1690 1700 1710 1720 1730 1740 1750

---------+---------+---------+---------+---------+---------+---------+-----

DLS11685_ORF1.pro ..D...........R..............A............................I.......S..T..V.

KJ701409_ORF1.pro ..............G.........................................................V.

KU176129_ORF1.pro ..............G.........................................................V.

AB248520_ORF1.pro ..............R................E..........................................

AB291962_ORF1.pro ..............G...........................................I.............V.

DLS11677_ORF1.pro ..............G.........................................................V.

DLS11681_ORF1.pro ..............G........................................I................E.

JQ953664_ORF1.pro ..............G.........................................................V.

AB073912_ORF1.pro ..............G...........................................I.............V..

AB189070_ORF1.pro ..............G...........................................I.............V.

AB248521_ORF1.pro ..............R......................................................T....

AB290312_ORF1.pro ..............G..................................................S......V.

AB290313_ORF1.pro ..............G............P..............................................

AB369687_ORF1.pro ..............G...........................................................

AB369689_ORF1.pro ..............R...........................................I.............V.

AF060668_ORF1.pro ..............G...........................................I.............V.

AF082843_ORF1.pro ..............G...........................................I.............V.

AF455784_ORF1.pro ..............G................E..........................................

AP003430_ORF1.pro ..............G..........................................SI.............V.

AY115488_ORF1.pro ..............R...........................................I.............V.

EU360977_ORF1.pro ..X...........G...........................................................

EU723513_ORF1.pro ..............R...........................................................

FJ705359_ORF1.pro ..............G.........................................................V.

FJ998008_ORF1.pro ..............G.........................................................V.

JQ013794_ORF1.pro ..............G.........................................................I.

AB740220_ORF1.pro ......................................................Y............T.M....

AB740221_ORF1.pro ......................................................Y..............V....

AB740222_ORF1.pro ......................................................Y..............V....

FJ906895_ORF1.pro ......................................................Y..............V....

FJ906896_ORF1.pro .......................A..............................Y..............V....

GU937805_ORF1.pro ......................................................Y..............V....

JQ013791_ORF1.pro .............................A........................Y..S................

JQ013792_ORF1.pro ......................................................Y..S................

JQ013793_ORF1.pro ......................................................Y..............V....

JQ768461_ORF1.pro ...................V..................................Y..............V....

JX109834_ORF1.pro ...................V..................................Y..............V....

JX121233_ORF1.pro ......................................................Y..............V....

JX565469_ORF1.pro ......................................................Y..............V....

KJ013414_ORF1.pro ......................................................Y..............V....

KJ013415_ORF1.pro ...............................XG.....................Y..............V....

KX227751_ORF1.pro ......................................................Y..............V....

KY436898_ORF1.pro ......................................................Y...................

KY496200_ORF1.pro .............................A........................Y..............V....

MF480297_ORF1.pro ..G...................................................Y..............VY.I.

MF480298_ORF1.pro ......................................................Y..............L....

MG211750_ORF1.pro ..............R...........................................................

MG211751_ORF1.pro ..............R...........................................................

Note 1: Amino acid sequences of rabbit HEV strains, DLS13-11685, and DLS13-11677 and DLS13-11681 are highlighted in yellow, blue, and green, respectively. Amino acids in the sequence of DLS13-11685 that appear exclusively (yellow) or predominantly (red) in the sequences of rabbit HEV but not in HEV-3 strains are highlighted.

Note 2: The breakpoints of nucleotides (nt) 38 and 891 for fragment 1 (Fig 3A), and 4794 for fragment 2 (Fig 3B) are indicated above the majority sequence. The positions of the amino acids at the breakpoints are also annotated.
