# Supplementary material for: Identification of a putative novel genotype 3/rabbit hepatitis E virus (HEV) recombinant
Source: PLoS One. 2018 Sep 11;13(9):e0203618. doi: 10.1371/journal.pone.0203618 (PMC6133284; doi:10.1371/journal.pone.0203618)
Supplement: S5 Table — (DOCX) [file pone.0203618.s005.docx]

**S5 Table.** Alignment of the amino acid sequences of the ORF2-encoded proteins of 22 HEV-3 strains, 22 rabbit HEV strains, and three novel strains (DLS13-11677, DLS13-11681 and DLS13-11685).

nt 5157 (aa 7)

Majority MRPRAVLLLFLVFLPMLPAPPAGQPSGRRRGRRSGGAGGGFWGDRVDSQPFALPYIHPTNPFAADVVSQSGAGARPRQPA

---------+---------+---------+---------+---------+---------+---------+---------+

10 20 30 40 50 60 70 80

---------+---------+---------+---------+---------+---------+---------+---------+

KU176129_ORF2.pro ....................................T.S............................P...........P

AB248520_ORF2.pro ..........F.L........................S.............................P.P.........P

AB291962_ORF2.pro ..........F.L........................................................P.........P

DLS11677_ORF2.pro ............L............P..........T.S.......................V...I....V.......P

DLS11681_ORF2.pro ............L.......................T.S.......................V...I....V.......P

DLS11685_ORF2.pro .....L...L............................S...........................LP.P........SP

KJ701409_ORF2.pro ............L........T...X..........T.S......A....................I..P...T.....P

JQ953664_ORF2.pro ..........F.L............T..........T.S...........................I..P.....S...P

AB073912_ORF2.pro .C.......LF.L.....................................................F............P

AB189070_ORF2.pro ..........F.L.........................S..................................V.....P

AB248521_ORF2.pro ..........F.L......................S.................................P.....S...P

AB290312_ORF2.pro .........................T...........SS............................P..........SP

AB290313_ORF2.pro ..........F.L......................S.......................................S..SV

AB369687_ORF2.pro ..........F.L........................................................P..........

AB369689_ORF2.pro ....................................T..........................................P

AF060668_ORF2.pro ...........M..............................S..........................P...T.....P

AF082843_ORF2.pro .........LF.L................C...N...................................P...V.....P

AF455784_ORF2.pro .C..........L................C......T.............................I..P........AP

AP003430_ORF2.pro .........LF.L..................................................................P

AY115488_ORF2.pro .....I....F.L..................................................T.........T.....P

EU360977_ORF2.pro ..H.................................S...........................................

EU723513_ORF2.pro .C..V.....F.............S......................................S..L..P..........

FJ705359_ORF2.pro ..........F...........................S............................P...........S

FJ998008_ORF2.pro ............L............A............S........................S.....P.........P

JQ013794_ORF2.pro .........................T...........................................P.........P

AB740220_ORF2.pro ...G.....L..L.........S.ST.......G..SX.................F..........EPH..S..K.....

AB740221_ORF2.pro ...G.I...LV.C............A.......GS.P..................F..........DPHP.S........

AB740222_ORF2.pro ..TGV....L..............ST.......G..S..................F..........D.H..S..K.....

FJ906895_ORF2.pro ..TGV....L..............ST.......G..S..S...............F..........D.H..S........

FJ906896_ORF2.pro ..SG........C...........SA.......GS.P.S................F..........DPHP.S.......T

GU937805_ORF2.pro ...G.I....V.C............AS......GS.P.S................F..........D.H..S........

JQ013791_ORF2.pro ..L.F....LF......................G.ST.S................F..........DTH..S...A....

JQ013792_ORF2.pro .CL.SI....FML.......................T..................L..........DTH..S........

JQ013793_ORF2.pro ...G.I...L..C............A..........S........I.........L.......T..DPH..S.T......

JQ768461_ORF2.pro ...G.I....V.C............AS......GS.P.S................F..........H.H..S........

JX109834_ORF2.pro ...G.I....V.C............AS......GS.P.S................F..........H.H..S........

JX121233_ORF2.pro ...G.I....V.C............AS......GS.P.S................F..........H.H..S........

JX565469_ORF2.pro ..SG.....LF.CM..........STA......G..S..................F....T.....D.H..S.....H..

KJ013414_ORF2.pro ...G.I....V..............AS......GS.P.S................F..........H.H..S........

KJ013415_ORF2.pro ...G.I....V..............AS......GS.P.S................F..........H.H..S........

KX227751_ORF2.pro ..LG.....L............S..T.......G..S..................F..........D.HP.S..K.....

KY436898_ORF2.pro .........LF..............A.......G..S.S................F..........D.H..S......S.

KY496200_ORF2.pro .........LF.C...........SA.......G..P..................F..........D.HP.P........

MF480297_ORF2.pro .CS..I...L...............A.......N..T.S......A.........F..........N.HP.S........

MF480298_ORF2.pro .....I...LF.............SA.......G.SS.S................F..........D.H..S...S....

MG211750_ORF2.pro ...G.L...................A.......G..S.S................L..........DTHP.S.......T

MG211751_ORF2.pro ...G.L...................A.......G..S.S................L..........DTHP.S.......T

nt 5584 (aa 149)

Majority RP-LGSSWRDQSQRPSAAPRRRSTPAGAAPLTAVSPAPDTAPVPDVDSRGAILRRQYNLSTSPLTSSVASGTNLVLYAAP

---------+---------+---------+---------+---------+---------+---------+---------+

90 100 110 120 130 140 150 160

---------+---------+---------+---------+---------+---------+---------+---------+

KU176129_ORF2.pro ..-..............V.....A........................................................

AB248520_ORF2.pro ..-...............S.............................................................

AB291962_ORF2.pro ..-...A.........V.S....A.T.......I..............................................

DLS11677_ORF2.pro P.-P................S..D.T......................................................

DLS11681_ORF2.pro ..-....................A.T.......T..............................................

DLS11685_ORF2.pro ..P............A.......AT..............AV.......H.................T.............

KJ701409_ORF2.pro ..-.............V......A.T.......I..............................................

JQ953664_ORF2.pro ..-...T........P.T.....A........................................................

AB073912_ORF2.pro ..-...A..........................T..............................................

AB189070_ORF2.pro ..-...A........P.......A.........I..............................................

AB248521_ORF2.pro ..-............P................................................................

AB290312_ORF2.pro ..-...T.........V.S....A........................................................

AB290313_ORF2.pro ..-.............................................................................

AB369687_ORF2.pro ..-....................A.T......................................................

AB369689_ORF2.pro ..-...A................A.........T..............................................

AF060668_ORF2.pro ..-...A.....K...V................I..............................................

AF082843_ORF2.pro ..-...A.........T......A.............................................A..........

AF455784_ORF2.pro ..-.............................................................................

AP003430_ORF2.pro ..-...A........T......PA.........T..............................................

AY115488_ORF2.pro ..-...A........P.VS....A.........T..............................................

EU360977_ORF2.pro ..-............P.........T.............S........................................

EU723513_ORF2.pro ..-............P.........T......................................................

FJ705359_ORF2.pro ..-..............V.....A.........I..............................................

FJ998008_ORF2.pro ..-....................A.T.......I..............................................

JQ013794_ORF2.pro ..-...T...........F....A.........X.................................I............

AB740220_ORF2.pro ..-..T....L.......S....A.T........................................T.............

AB740221_ORF2.pro ..-..T....L....P..S...............................................T.............

AB740222_ORF2.pro ..-..T....L....P..S...............................................T.............

FJ906895_ORF2.pro ..-..T....L....P..S......T........................................T.............

FJ906896_ORF2.pro ..-..T....L....P.........T........................................T..A..........

GU937805_ORF2.pro ..-..T....L....P..S...............................................T.............

JQ013791_ORF2.pro ..-.......L....P.GS....A................V.......................................

JQ013792_ORF2.pro ..-.......L....P.G.......T......................................................

JQ013793_ORF2.pro ..-..T....L.......S...TA.T........................................T.............

JQ768461_ORF2.pro ..-..T....L....P..S...............................................T.............

JX109834_ORF2.pro ..-..T....L....P..S...............................................T.............

JX121233_ORF2.pro ..-..T....L....P..S...............................................T.............

JX565469_ORF2.pro ..-..T....L....P..S......T......................................LLT.............

KJ013414_ORF2.pro ..-..T....L....P..S...............................................T.............

KJ013415_ORF2.pro ..-..T....L....P..S...............................................T.............

KX227751_ORF2.pro ..-..T....L....P..S......T........................................T.............

KY436898_ORF2.pro ..-..T....L....P.P.......T........................................T.............

KY496200_ORF2.pro ..-..T....L............A..........................................T.............

MF480297_ORF2.pro ..-.......L......T.....A.T........................................TI............

MF480298_ORF2.pro ..-..T....L....T.T....PA................V.........................T.............

MG211750_ORF2.pro ..-..T....L......TS...............................................T.............

MG211751_ORF2.pro ..-..T....L......TS...............................................T.............

Majority LNPLLPLQDGTNTHIMATEASNYAQYRVVRATIRYRPLVPNAVGGYAISISFWPQTTTTPTSVDMNSITSTDVRILVQPG

---------+---------+---------+---------+---------+---------+---------+---------+

170 180 190 200 210 220 230 240

---------+---------+---------+---------+---------+---------+---------+---------+

KU176129_ORF2.pro ................................................................................

AB248520_ORF2.pro ................................................................................

AB291962_ORF2.pro ................................................................................

DLS11677_ORF2.pro ................................................................................

DLS11681_ORF2.pro ................................................................................

DLS11685_ORF2.pro ...............................................V................................

KJ701409_ORF2.pro ............................X...................................................

JQ953664_ORF2.pro ................................................................................

AB073912_ORF2.pro ................................................................................

AB189070_ORF2.pro ............................A...................................................

AB248521_ORF2.pro ................................................................................

AB290312_ORF2.pro ................................................................................

AB290313_ORF2.pro ................................................................................

AB369687_ORF2.pro ................................................................................

AB369689_ORF2.pro ................................................................................

AF060668_ORF2.pro ................................................................................

AF082843_ORF2.pro ................................................................................

AF455784_ORF2.pro ................................................................................

AP003430_ORF2.pro ................................................................................

AY115488_ORF2.pro ................................................................................

EU360977_ORF2.pro ................................................................................

EU723513_ORF2.pro ................................................................................

FJ705359_ORF2.pro ................................................................................

FJ998008_ORF2.pro ................................................................................

JQ013794_ORF2.pro ................................................................................

AB740220_ORF2.pro ................................................................................

AB740221_ORF2.pro ................................................................................

AB740222_ORF2.pro ................................................................................

FJ906895_ORF2.pro ..............................................................I.................

FJ906896_ORF2.pro ......................................................................A.........

GU937805_ORF2.pro ................................................................................

JQ013791_ORF2.pro ...............................................V................................

JQ013792_ORF2.pro ............................A..................V................................

JQ013793_ORF2.pro ................................................................................

JQ768461_ORF2.pro .I..............................................................................

JX109834_ORF2.pro .I..............................................................................

JX121233_ORF2.pro ................................................................................

JX565469_ORF2.pro ........................................VT......................................

KJ013414_ORF2.pro ................................................................................

KJ013415_ORF2.pro ................................................................................

KX227751_ORF2.pro ................................................................................

KY436898_ORF2.pro ................................................................................

KY496200_ORF2.pro ................................................................................

MF480297_ORF2.pro ............................A..................V................................

MF480298_ORF2.pro ................................................................................

MG211750_ORF2.pro ................................................................................

MG211751_ORF2.pro ................................................................................

Majority XASELVIPSERLHYRNQGWRSVETSGVAEEEATSGLVMLCIHGSPVNSYTNTPYTGALGLLDFALELEFRNLTPGNTNTR

---------+---------+---------+---------+---------+---------+---------+---------+

250 260 270 280 290 300 310 320

---------+---------+---------+---------+---------+---------+---------+---------+

KU176129_ORF2.pro I...............................................................................

AB248520_ORF2.pro V...............................................................................

AB291962_ORF2.pro I.......................T.......................................................

DLS11677_ORF2.pro I...............................................................................

DLS11681_ORF2.pro I...............................................................................

DLS11685_ORF2.pro L...................................I...........................................

KJ701409_ORF2.pro I...............................................................................

JQ953664_ORF2.pro I...............................................................................

AB073912_ORF2.pro I.......................T.......................................................

AB189070_ORF2.pro I.......................T.......................................................

AB248521_ORF2.pro I...............................................................................

AB290312_ORF2.pro I...............................................................................

AB290313_ORF2.pro I...............................................................................

AB369687_ORF2.pro I...............................................................................

AB369689_ORF2.pro I.......................T.......................................................

AF060668_ORF2.pro I.......................T.......................................................

AF082843_ORF2.pro I.......................T.......................................................

AF455784_ORF2.pro I...............................................................................

AP003430_ORF2.pro I.......................T.......................................................

AY115488_ORF2.pro I.......................T.......................................................

EU360977_ORF2.pro I...............................................................................

EU723513_ORF2.pro I...............................................................................

FJ705359_ORF2.pro I...............................................................................

FJ998008_ORF2.pro I...............................................................................

JQ013794_ORF2.pro I...............................................................................

AB740220_ORF2.pro L.................................................................I.............

AB740221_ORF2.pro L.................................................................I.............

AB740222_ORF2.pro L.................................................................I.............

FJ906895_ORF2.pro L.................................................................V.............

FJ906896_ORF2.pro L.................G-ALWRPL.CR.RPLP..SCFAPW-L.C....................I.............

GU937805_ORF2.pro L.................................................................I.............

JQ013791_ORF2.pro L...............................................................................

JQ013792_ORF2.pro L...............................................................................

JQ013793_ORF2.pro L.................................................................I.............

JQ768461_ORF2.pro L.................................................................I.............

JX109834_ORF2.pro L.................................................................I.............

JX121233_ORF2.pro L.................................................................I.............

JX565469_ORF2.pro L.................................................................I.............

KJ013414_ORF2.pro L.................................................................I.............

KJ013415_ORF2.pro L...........Y.....................................................I.............

KX227751_ORF2.pro L.................................................................I.............

KY436898_ORF2.pro L.................................................................I.............

KY496200_ORF2.pro L............................K....................................I.............

MF480297_ORF2.pro L.................................................................I.............

MF480298_ORF2.pro L.................................................................I.............

MG211750_ORF2.pro L.................................................................I.............

MG211751_ORF2.pro L.............X...................................................I.............

Majority VSRYTSTARHRLRRGADGTAELTTTAATRFMKDLHFTGTNGVGEVGRGIALTLFNLADTLLGGLPTELISSAGGQLFYSR

---------+---------+---------+---------+---------+---------+---------+---------+

330 340 350 360 370 380 390 400

---------+---------+---------+---------+---------+---------+---------+---------+

KU176129_ORF2.pro ................................................................................

AB248520_ORF2.pro ................................................................................

AB291962_ORF2.pro ................................................................................

DLS11677_ORF2.pro ................................................................................

DLS11681_ORF2.pro ................................................................................

DLS11685_ORF2.pro ......................................M...........M........................V....

KJ701409_ORF2.pro ........................................................X.......................

JQ953664_ORF2.pro ................................................................................

AB073912_ORF2.pro ................................................................................

AB189070_ORF2.pro ................................................................................

AB248521_ORF2.pro ...............P................................................................

AB290312_ORF2.pro ................................................................................

AB290313_ORF2.pro ................................................................................

AB369687_ORF2.pro ................................................................................

AB369689_ORF2.pro ................................................................................

AF060668_ORF2.pro ................................................................................

AF082843_ORF2.pro ................................................................................

AF455784_ORF2.pro ................................................................................

AP003430_ORF2.pro ................................................................................

AY115488_ORF2.pro ................................................................................

EU360977_ORF2.pro ................................................................................

EU723513_ORF2.pro ................................................................................

FJ705359_ORF2.pro ................................................................................

FJ998008_ORF2.pro ................................................................................

JQ013794_ORF2.pro ................................................................................

AB740220_ORF2.pro ......................................M.....I..........I...................V....

AB740221_ORF2.pro ......................................M.....I..............................V....

AB740222_ORF2.pro ......................................M.....I..........I...................V....

FJ906895_ORF2.pro ......................................M.....I..........I...................V....

FJ906896_ORF2.pro ......................................M.....I..............................V....

GU937805_ORF2.pro ......................................M.....I.....P........................I....

JQ013791_ORF2.pro ......................................M.....I...................................

JQ013792_ORF2.pro ......................................M.....I...................................

JQ013793_ORF2.pro ............................................I..............................V....

JQ768461_ORF2.pro ......................................M.....I..............................V....

JX109834_ORF2.pro ......................................M.....I..............................V....

JX121233_ORF2.pro ......................................M.....I..............................V....

JX565469_ORF2.pro ......................................M.....I..........I...................V....

KJ013414_ORF2.pro ......................................M.....I..............................V....

KJ013415_ORF2.pro ......................................M.....I..............................V....

KX227751_ORF2.pro ......................................M.....I..............................V....

KY436898_ORF2.pro ............................................I..............................V....

KY496200_ORF2.pro ......................................M.....I..............................V....

MF480297_ORF2.pro ....S.................................M.....I..........I..............AV...AL...

MF480298_ORF2.pro ..................................Y...M.....I..............................V....

MG211750_ORF2.pro ......................................M.....I..............................V....

MG211751_ORF2.pro ......................................M.....I..........X...................V....

Majority PVVSANGEPTVKLYTSVENAQQDKGIAIPHDIDLGDSRVVIQDYDNQHEQDRPTPSPAPSRPFSVLRANDVLWLSLTAAE

---------+---------+---------+---------+---------+---------+---------+---------+

410 420 430 440 450 460 470 480

---------+---------+---------+---------+---------+---------+---------+---------+

KU176129_ORF2.pro ................................................................................

AB248520_ORF2.pro ................................................................................

AB291962_ORF2.pro ..........................T.....................................................

DLS11677_ORF2.pro .........A......................................................................

DLS11681_ORF2.pro .........A......................................................................

DLS11685_ORF2.pro ...............................................................F.............G.N

KJ701409_ORF2.pro .........A.............X........................................................

JQ953664_ORF2.pro .........A......................................................................

AB073912_ORF2.pro ..........................T.....................................................

AB189070_ORF2.pro ..........................T.....................................................

AB248521_ORF2.pro ................................................................................

AB290312_ORF2.pro ................................................................................

AB290313_ORF2.pro ................................................................................

AB369687_ORF2.pro ................................................................................

AB369689_ORF2.pro ..........................T.....................................................

AF060668_ORF2.pro ..........................T.....................................................

AF082843_ORF2.pro ..........................T.....................................................

AF455784_ORF2.pro ................................................................................

AP003430_ORF2.pro ..........................T.....................................................

AY115488_ORF2.pro ..........................T.....................................................

EU360977_ORF2.pro ................................................................................

EU723513_ORF2.pro ................................................................................

FJ705359_ORF2.pro ........................................V.......................................

FJ998008_ORF2.pro ................................................................................

JQ013794_ORF2.pro .......................................M........................................

AB740220_ORF2.pro .........................................................................V......

AB740221_ORF2.pro ................................................................................

AB740222_ORF2.pro .........................................................................V......

FJ906895_ORF2.pro ...................................................Q.....................V......

FJ906896_ORF2.pro .........A.....P...........L............L.......................................

GU937805_ORF2.pro ................................................................................

JQ013791_ORF2.pro .........A......................................................................

JQ013792_ORF2.pro ................................................................................

JQ013793_ORF2.pro ................................................................................

JQ768461_ORF2.pro ...................................................................V............

JX109834_ORF2.pro ...................................................................V............

JX121233_ORF2.pro ...................................................................V............

JX565469_ORF2.pro ..........................T..............................................V......

KJ013414_ORF2.pro ...................................................................V............

KJ013415_ORF2.pro ...................................................................V............

KX227751_ORF2.pro ................................................................................

KY436898_ORF2.pro ................................................................................

KY496200_ORF2.pro ................................................................................

MF480297_ORF2.pro ..P.SY.N....I...LD...H............................H.............................

MF480298_ORF2.pro ................................................................................

MG211750_ORF2.pro ................................................................................

MG211751_ORF2.pro ................................................................................

Majority YDQTTYGSSTNPMYVSDTVTFVNVATGAQAVARSLDWSKVTLDGRPLTTIQQYSKTFYVLPLRGKLSFWEAGTTKAGYPY

---------+---------+---------+---------+---------+---------+---------+---------+

490 500 510 520 530 540 550 560

---------+---------+---------+---------+---------+---------+---------+---------+

KU176129_ORF2.pro ................................................................................

AB248520_ORF2.pro .................................................V..............................

AB291962_ORF2.pro ................................................................................

DLS11677_ORF2.pro ................................................................................

DLS11681_ORF2.pro ................................................................................

DLS11685_ORF2.pro .....H..........................................I.E.N..SY.......................

KJ701409_ORF2.pro ................................................................................

JQ953664_ORF2.pro ....................L...........................................................

AB073912_ORF2.pro ................................................................................

AB189070_ORF2.pro ................................................................................

AB248521_ORF2.pro ................................................................................

AB290312_ORF2.pro ................................................................................

AB290313_ORF2.pro ............................................................L...................

AB369687_ORF2.pro ................................................................................

AB369689_ORF2.pro ....................L...........................................................

AF060668_ORF2.pro .X..................L..................................K........................

AF082843_ORF2.pro ....................L...........................................................

AF455784_ORF2.pro ................................................................................

AP003430_ORF2.pro ................................................................................

AY115488_ORF2.pro .................................................T...............M..............

EU360977_ORF2.pro ................................................................................

EU723513_ORF2.pro ................................................................................

FJ705359_ORF2.pro ................................................................................

FJ998008_ORF2.pro ................................................................................

JQ013794_ORF2.pro ......................................R.............X...........................

AB740220_ORF2.pro ....................................................H..I........................

AB740221_ORF2.pro ....................................................H..M........................

AB740222_ORF2.pro .....................................A..............H..L........................

FJ906895_ORF2.pro ....................................................H..I........................

FJ906896_ORF2.pro ....................................................H..M........................

GU937805_ORF2.pro ................N...................................HF..........................

JQ013791_ORF2.pro ...A........V.I................................I................................

JQ013792_ORF2.pro ...S........V................................Q..................................

JQ013793_ORF2.pro ..................................................L.H...........................

JQ768461_ORF2.pro ....................................................H...........................

JX109834_ORF2.pro ....................................................H...........................

JX121233_ORF2.pro ....................................................H...........................

JX565469_ORF2.pro .....................................T..............H..I........................

KJ013414_ORF2.pro ....................................................H...........................

KJ013415_ORF2.pro ....................................................H...........................

KX227751_ORF2.pro ....................................................H...........................

KY436898_ORF2.pro ....................................................H...........................

KY496200_ORF2.pro ....................................................H...........................

MF480297_ORF2.pro ...SV.......L........................A............E.H...........................

MF480298_ORF2.pro ...SV.......L........................A............E.H...........................

MG211750_ORF2.pro ....DF......L...............................Q.......H.R...I.....................

MG211751_ORF2.pro ....DF..X...L...............................Q.......H.R...I.....................

Majority NYNTTASDQILIENAAGHRVAISTYTTSLGAGPVSISAVGVLAPHSALAVLEDTIDYPARAHTFDDFCPECRTLGLQGCA

---------+---------+---------+---------+---------+---------+---------+---------+

570 580 590 600 610 620 630 640

---------+---------+---------+---------+---------+---------+---------+---------+

KU176129_ORF2.pro ...................................V....................................A.......

AB248520_ORF2.pro ...................................V........................T...........N.......

AB291962_ORF2.pro .................................T....................V.................I.......

DLS11677_ORF2.pro ...........V.......................V..................A.........................

DLS11681_ORF2.pro ...................................V....................................A.......

DLS11685_ORF2.pro .........V.....S.YL........N.......................S............................

KJ701409_ORF2.pro ..D.......................P........V..................T.C......Y........A.......

JQ953664_ORF2.pro ...................................V.............I..............................

AB073912_ORF2.pro .................................T....................V.................A.......

AB189070_ORF2.pro ..D........V.....................T....................A.........................

AB248521_ORF2.pro ...................................V.............L......................N.......

AB290312_ORF2.pro ...........T.....R.................V.............I....T.................A.......

AB290313_ORF2.pro ...................................V..A..........M......................N.......

AB369687_ORF2.pro ...................................V...................X................N.......

AB369689_ORF2.pro .................................T..............................................

AF060668_ORF2.pro .................................T....................V.........................

AF082843_ORF2.pro .................................T....................V.........................

AF455784_ORF2.pro ...................................V.............A......................N.......

AP003430_ORF2.pro .................................T....................A.........................

AY115488_ORF2.pro .................................T....................V.........................

EU360977_ORF2.pro ...................................V.............M....T.................N.......

EU723513_ORF2.pro ...................................V....................................N.......

FJ705359_ORF2.pro ...................................V....................................A.......

FJ998008_ORF2.pro ......................................................V.................A.......

JQ013794_ORF2.pro ...................................V.............I....V.................A.......

AB740220_ORF2.pro ...............TD....V........................V.......V.........................

AB740221_ORF2.pro ................D....V........................V.........................I.......

AB740222_ORF2.pro ................D....V........................V.......V.........................

FJ906895_ORF2.pro ...PP...........D....V...P.......A..........P.V...F...V............R............

FJ906896_ORF2.pro ................D....V....A.........F.........V...................S.............

GU937805_ORF2.pro ................D....V.............F..........V.........................I.......

JQ013791_ORF2.pro ....V...................................................................A.......

JQ013792_ORF2.pro ....V.........G...............................V.........................V.......

JQ013793_ORF2.pro ................D....V........................V.......V.........................

JQ768461_ORF2.pro ................D....V........................V.........................I.......

JX109834_ORF2.pro ................D....V........................V.........................I.......

JX121233_ORF2.pro ................D....V........................V.........................I.......

JX565469_ORF2.pro ................E....V........................V.......V.........................

KJ013414_ORF2.pro ................D....V........................V.........................I.......

KJ013415_ORF2.pro ................D....V........................V.........................I.......

KX227751_ORF2.pro ................D....V........................V.......V.................I.......

KY436898_ORF2.pro ................D....V........................V.................................

KY496200_ORF2.pro ................D.H..V........................V.................................

MF480297_ORF2.pro ....V......V...SD.............................V....................F.AAVLF..P.R.

MF480298_ORF2.pro ....V......V...SD.............................V.........................I.......

MG211750_ORF2.pro ..X............T...................V..........V.........................A.......

MG211751_ORF2.pro ...............T...................V..........V.........................A.......

nt 7093 (aa 652)

Majority FQSTVAELQRLKMKVGKTRES

---------+---------+-

650 660

---------+---------+-

KU176129_ORF2.pro ....I...............F

AB248520_ORF2.pro ....I................

AB291962_ORF2.pro ....I................

DLS11677_ORF2.pro ....I...............F

DLS11681_ORF2.pro ....I...............F

DLS11685_ORF2.pro .....................

KJ701409_ORF2.pro ....I.......

JQ953664_ORF2.pro ....I................

AB073912_ORF2.pro .....................

AB189070_ORF2.pro .....................

AB248521_ORF2.pro ....I................

AB290312_ORF2.pro ....I...............F

AB290313_ORF2.pro ....I................

AB369687_ORF2.pro ....I................

AB369689_ORF2.pro .....................

AF060668_ORF2.pro ....I................

AF082843_ORF2.pro ....I................

AF455784_ORF2.pro ....I................

AP003430_ORF2.pro ....I...............F

AY115488_ORF2.pro ....I................

EU360977_ORF2.pro ....I................

EU723513_ORF2.pro .....................

FJ705359_ORF2.pro ....I.........L.....F

FJ998008_ORF2.pro ....I...............F

JQ013794_ORF2.pro ....I...............F

AB740220_ORF2.pro .....................

AB740221_ORF2.pro .....................

AB740222_ORF2.pro ....................F

FJ906895_ORF2.pro ..F.................F

FJ906896_ORF2.pro ....................F

GU937805_ORF2.pro .....................

JQ013791_ORF2.pro ............L........

JQ013792_ORF2.pro .....................

JQ013793_ORF2.pro ............V........

JQ768461_ORF2.pro .....................

JX109834_ORF2.pro .....................

JX121233_ORF2.pro .....................

JX565469_ORF2.pro ....................F

KJ013414_ORF2.pro .....................

KJ013415_ORF2.pro .....................

KX227751_ORF2.pro .....................

KY436898_ORF2.pro .....................

KY496200_ORF2.pro .....................

MF480297_ORF2.pro ....DTVAHP..K........

MF480298_ORF2.pro .....................

MG211750_ORF2.pro .....................

MG211751_ORF2.pro .....................

Note 1: Amino acid sequences of rabbit HEV strains, DLS13-11685, and DLS13-11677 and DLS13-11681 are highlighted in yellow, blue, and green, respectively. Amino acids in the sequence of DLS13-11685 that appear exclusively (yellow) or predominantly (red) in the sequences of rabbit HEV but not in HEV-3 strains are highlighted.

Note 2: The breakpoints of nucleotides (nt) 5157 for fragment 3 (Fig 3C), 5584 for fragment 4 (Fig 3D), and 7093 for fragment 5 (Fig 3E) are indicated above the majority sequence. The positions of the amino acids at the breakpoints are also annotated.
