# Supplementary material for: Identification of a putative novel genotype 3/rabbit hepatitis E virus (HEV) recombinant
Source: PLoS One. 2018 Sep 11;13(9):e0203618. doi: 10.1371/journal.pone.0203618 (PMC6133284; doi:10.1371/journal.pone.0203618)
Supplement: S6 Table — (DOCX) [file pone.0203618.s006.docx]

**S6 Table.** Alignment of the amino acid sequences of the ORF3-encoded proteins of 22 HEV-3 strains, 22 rabbit HEV strains, and three novel strains (DLS13-11677, DLS13-11681 and DLS13-11685).

5157 nt (aa 21)

\\\

Majority MNNMF-CASPMGS-PCALGLFCCCSSCFCLCCPRHRPVSRLAAVVGGAAAVPAVVSGVTGLILSPSPSPIFIQPTPSPPT

---------+---------+---------+---------+---------+---------+---------+---------+

10 20 30 40 50 60 70 80

---------+---------+---------+---------+---------+---------+---------+---------+

AB248520_ORF3.pro ....L-.......-.......................A......................................L...

AB291962_ORF3.pro ....S-.......-.......................A......A..................................M

AF051830_ORF3.pro ....S-F.A.T..RS...................................................Q............M

DLS11677_ORF3.pro .....-.......-.......................A......................................LS.M

DLS11681_ORF3.pro .....-.......-.......................A......................................LS.M

DLS11685_ORF3.pro ....S-..L....-......Y...............................V..........................M

KJ701409_ORF3.pro .....-.......-.......................A......................................L..M

KU176129_ORF3.pro .....-.......-.......................A......................................L..M

JQ953664_ORF3.pro ....S-..L....-.......................A......A...............................L...

AB189070_ORF3.pro ....S-R......-.......................A.........................................M

AB248521_ORF3.pro ....L-.......-.......................A......................................L...

AB290312_ORF3.pro ....S-..L....-.......................A......A..................................M

AB290313_ORF3.pro .....-.......-S......................A......A...............................L...

AB369687_ORF3.pro .....-.......-.......................A..........................................

AB369689_ORF3.pro ....S-.......-.......................A......A..................................M

AF060668_ORF3.pro ....S-F......-............................VA...................................M

AF082843_ORF3.pro ....S-F......-.......................A..........T............................L.M

AF455784_ORF3.pro ....S-.......-.......................A......................................L...

AP003430_ORF3.pro ....S-.......-.......................A.........................................M

AY115488_ORF3.pro ....S-......P-.......................A....V....................................M

EU360977_ORF3.pro .....-..L....-...I..........................................................L...

EU723513_ORF3.pro .....-.......-.......................A......................................L...

FJ705359_ORF3.pro .....-..L....-.......................A......................................L..M

FJ998008_ORF3.pro .....-..L....-.......................A....V...................P.............L..M

JQ013794_ORF3.pro .....-.......-.......................A.........................................M

AB740220_ORF3.pro .....-S..L...-................................................................L.

AB740221_ORF3.pro .....FF......-.............V..................................................L.

AB740222_ORF3.pro .....-S......-................................................................L.

FJ906895_ORF3.pro ...T.-S......-................................................................L.

FJ906896_ORF3.pro ....S-F..L...-.............V..................................................L.

GU937805_ORF3.pro .....LF......-.............V..................................................L.

JQ013791_ORF3.pro ....S-S......-..................................................................

JQ013792_ORF3.pro .....-S......-..................................................................

JQ013793_ORF3.pro ....S-S......-.............V..................................................L.

JQ768461_ORF3.pro .....LF......-.............V..................................................L.

JX109834_ORF3.pro .....LF......-.............V..................................................L.

JX121233_ORF3.pro .....LF......-.............V..................................................L.

JX565469_ORF3.pro ...T.-S......-.............V.............P....................................L.

KJ013414_ORF3.pro .....LF......-................................................................L.

KJ013415_ORF3.pro .....LF......-.............................A..................................L.

KX227751_ORF3.pro ....S-S..L...-................................................................L.

KY436898_ORF3.pro .....-S.L....-................................................................L.

KY496200_ORF3.pro .....-S......-.............V..................................................L.

MF480297_ORF3.pro .....-S......-..................................T...............................

MF480298_ORF3.pro ----------...-..................................................................

MG211750_ORF3.pro ....S-F.P....-................................................................L.

MG211751_ORF3.pro ....S-F.P....-................................................................L.

Majority STHNPGLELALDSRPAP-LAPLGVTXPSAPPLPPVVDLPQLGLRR

---------+---------+---------+---------+-----

90 100 110 120

---------+---------+---------+---------+-----

AB248520_ORF3.pro .Y...............-S....A.S...................

AB291962_ORF3.pro .F...............-S......S...................

AF051830_ORF3.pro .PLR...D.VFANP.DH-S......R.......H........P..

DLS11677_ORF3.pro .FR.........NH.P.-PV.....S.......H....T...Q..

DLS11681_ORF3.pro .FR..............-.G.P...S.....P.H...........

DLS11685_ORF3.pro .Y...............PS......N...........Q.......

KJ701409_ORF3.pro .FR..........H...-S......S.......H...........

KU176129_ORF3.pro .YR..........H...-S....AIS......SH...........

JQ953664_ORF3.pro .F...............-SV.....S.....P..A..........

AB189070_ORF3.pro .F......S..G.....-.......S..............P....

AB248521_ORF3.pro .Y...............-S....A.S........A..........

AB290312_ORF3.pro LFR..........HH..-S......S...................

AB290313_ORF3.pro .YR...........S..-SV.....S........A..........

AB369687_ORF3.pro .Y...............-S......S...................

AB369689_ORF3.pro .F...............-.......S........A..........

AF060668_ORF3.pro .F...............-SV.....S...................

AF082843_ORF3.pro .F......F........-.......S...................

AF455784_ORF3.pro .F..........R....-S......S...................

AP003430_ORF3.pro .F...............-.....A.S...................

AY115488_ORF3.pro .F...............-.......S......S............

EU360977_ORF3.pro .Y..........N....-S....A.S.....P.............

EU723513_ORF3.pro .Y...............-S......S...................

FJ705359_ORF3.pro .YR..............-S.....IN......SH......P....

FJ998008_ORF3.pro .FR..............-.......S........A..........

JQ013794_ORF3.pro .F.........G.....-S......S.......S...........

AB740220_ORF3.pro LS.I....PNPG.Q...-.....A.C.......R...........

AB740221_ORF3.pro L..I....PDPGNQ...-.....A.C.......R...........

AB740222_ORF3.pro L..I....PNPGNQ...-.....A.C.......R...........

FJ906895_ORF3.pro L..I....PDPGNQ...-S.H..A.C.......R...........

FJ906896_ORF3.pro L..I....PDPG.Q...-.......C.......RA..........

GU937805_ORF3.pro ...I....PDPGNQ...-.......C.......RA..........

JQ013791_ORF3.pro L..I....PVPGN....-.......C......AHA..........

JQ013792_ORF3.pro L..I....PVPG.....-.V.P...C......A.A..........

JQ013793_ORF3.pro L..I....PVP..Q.V.-.....A.C.......R..E........

JQ768461_ORF3.pro F..I....PDPGNQ...-.......C.......RAA.........

JX109834_ORF3.pro F..I....PDPGNQ...-.......C.......RAA.........

JX121233_ORF3.pro F..I....PDPGNQ...-.......C.......RA..........

JX565469_ORF3.pro L..I....PDPGT....-..L....C.......RA..........

KJ013414_ORF3.pro F..I....PDPGNQ...-.......C.......RA..........

KJ013415_ORF3.pro F..I....PDPGNQ...-.......C.......RA..........

KX227751_ORF3.pro L..I....PNPG.Q...-.....A.C.......R...........

KY436898_ORF3.pro L..I....PVPG.Q...-.....A.C.......R.A.........

KY496200_ORF3.pro L..I....PDPV.Q...-.......C.......RA..........

MF480297_ORF3.pro L..I....P.HG.Q...-.....G.C.......RA..........

MF480298_ORF3.pro L..I....PVPG.Q...-.....A.C....Q..H...........

MG211750_ORF3.pro L..I....PVPG.Q.V.-SV...A.C....LP.HA..........

MG211751_ORF3.pro L..I....PVPG.Q.V.-SV...A.C....LP.HA..........

Note 1: Amino acid sequences of rabbit HEV strains, DLS13-11685, and DLS13-11677 and DLS13-11681 are highlighted in yellow, blue, and green, respectively.

Note 2: The breakpoint of nucleotide (nt) 5157 for fragment 3 (Fig 3C) is indicated above the majority sequence. The position of the amino acid at the breakpoint is also annotated.
