# Supplementary material for: Identification of a putative novel genotype 3/rabbit hepatitis E virus (HEV) recombinant
Source: PLoS One. 2018 Sep 11;13(9):e0203618. doi: 10.1371/journal.pone.0203618 (PMC6133284; doi:10.1371/journal.pone.0203618)
Supplement: S1 Fig — (DOCX) [file pone.0203618.s007.docx]

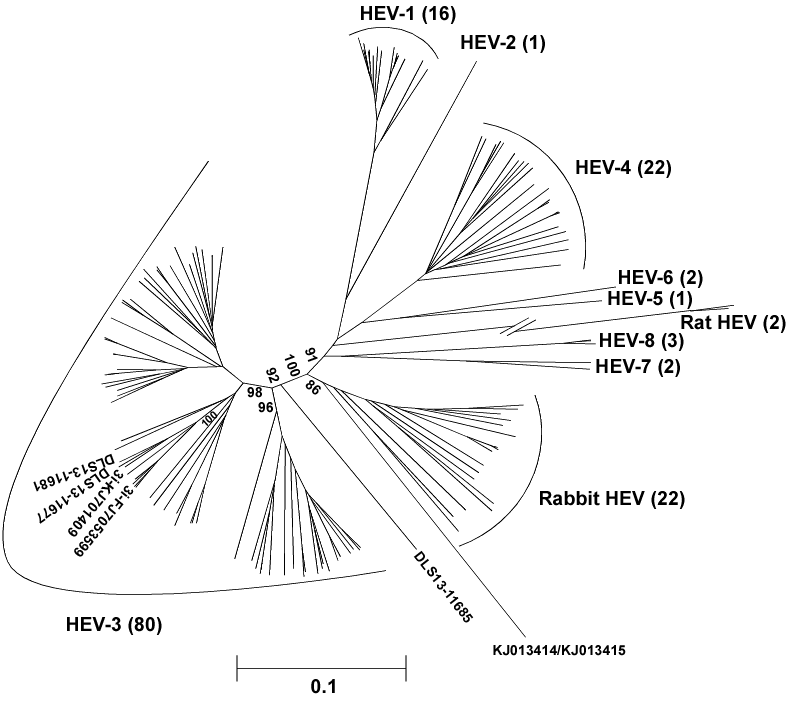


**S1 Fig. Phylogenetic tree of strains DLS13-11677, DLS13-11681 and DLS13-11685.** Phylogenetic tree was constructed by aligning complete genomes of 16 HEV-1, 1 HEV-2, 80 HEV-3, 22 rabbit HEV, 22 HEV-4, 1 HEV-5, 2 HEV-6, 2 HEV-7, 3 HEV-8, 2 rat HEV, DLS13-11677, DLS13-11681, and DLS13-11685. A total of 154 HEV sequences included the 81 HEV reference sequences listed in S2 Table and the 70 HEV reference sequences in S3 Table. The alignment was then gap-stripped to become 6,678 nucleotides long and converted to PHYLIP format using BioEdit Sequence Alignment Editor (version 5.0.9). Phylogenetic analysis was performed with the PHYLIP software package (version 3.5c). Phylogenetic tree was constructed using TreeExplorer software (version 2.12). Genotype designations for the reference strains are shown above the appropriate branches. Only the relevant GenBank accession numbers of the strains and bootstrap values are indicated in the tree.
